# Supramolecular Immobilization of Adamantyl and Carboxylate Modified *N*-Heterocyclic Carbene Ligand on Cucurbituril Substrates

**DOI:** 10.3390/molecules27051662

**Published:** 2022-03-03

**Authors:** Hamidou Keita

**Affiliations:** Department of Chemistry, Clemson University, Clemson, SC 29634, USA; hkeita@g.clemson.edu

**Keywords:** cucurbiturils, adamantane, non-covalent, homogeneous catalysts, host–guest chemistry, carboxylate, bidentate chelating NHC, benzimidazolium salts, immobilization

## Abstract

Herein, the design, synthesis, supramolecular interactions and structural analysis of a novel bidentate carboxylate chelating N-heterocylic carbene (NHC) ligand is presented. The NHC structure was modified to strategically incorporate adamantyl moiety for the formation of a supramolecular complex with host molecules such as cucurbiturils. The adamantyl modified NHC ligand could potentially be used in recoverable homogeneous catalysts when Immobilized on a solid support via host–guest chemistry. As a versatile precursor, NHC ligand (**8**) was synthesized and characterized by ^1^H-NMR, ^13^C-NMR, FTIR, single crystal x-ray crystallography and elemental analysis. A proof-of-principle non-covalent immobilization of the NHC ligand (**8**) with a Cucurbit[7]uril (CB7) host was demonstrated using ^1^H-NMR titration.

## 1. Introduction

The immobilization of homogeneous catalysts has been gaining traction in recent decades owing to their attractive features in recoverable and recyclable catalysts [1,2]. Key to this success is the sustained effort to obtain versatile ligands while ensuring their electronic properties are not negatively affected by modifications. Along this line, *N*-Heterocyclic carbenes (NHCs) ligands turn out to be the most appealing ligand for creative ligand designs and tuning various properties of the resulting catalyst for their industrial applications [3]. NHCs are a multifunctional group of ligands with impressive electronic properties; they serve as both an σ electron donor and a π electron acceptor which allows them to coordinate with metals while maintaining excellent stability compare to their phosphine counterparts [4,5,6].

The non-covalent immobilization of NHC compounds is an attractive strategy to reap the benefits of both the high efficiency of homogeneous catalysts and the ease of separation of heterogeneous catalysts [7]. To be specific, homogeneous catalysts are more efficient and selective than their heterogeneous counterparts. Heterogeneous catalysts are easier to separate and recycle, making them a prime choice over homogeneous catalysts for industrial applications. With noncovalent immobilizable NHC ligands, homogeneous catalysis and heterogeneous separation can be achieved simultaneously. The design of catalysts that can be recovered and reused provides sustainable alternatives for industrial applications and has amassed increased attention in recent years [8]. The design of such catalysts is typically achieved through the modification of the desired NHC ligands to afford varieties of functionalities including the ability to be immobilized on solid supports such as silica gel, polymer beads, carbon nanotubes and graphene [5,9].

While traditional heterogeneous catalysts are widely used in the industries, the process is plagued with low surface area and diminished catalytic activities, thereby limiting their applications. In recent years, novel solutions emerged in the form of non-covalent Immobilizable NHC ligands, where homogeneous catalysts are tethered to solid supports via π–π stacking [10], electrostatic [11], physio-sorption [2] or host–guest interactions [7]. They can be designed to include chemical moieties with superior supramolecular binding affinity and modular designs. A major challenge for catalysts that are immobilized via weaker forms of non-covalent interactions is leaching. However, this could be overcome by strategically designing a modular system where the NHC and the substrates are independently modified to obtain the desired binding affinity. Non-covalent immobilization methods have been reported in various catalysis such as olefin metathesis [7], hydrogenation [12], click reactions [1], amination reactions [13] and Pd-catalyzed cross-coupling reactions [14].

Unlike electrostatic interactions, which are susceptible to changes in pH and hence leaching of catalysts, host–guest interactions are a robust approach to non-covalent immobilization of NHC-based catalysts. In a recent study, adamantyl modified NHC ligand was tethered to β-cyclodextrin functionalized surface via host–guest interactions [7]. While tethering adamantane-modified catalysts onto β-cyclodextrin functionalized surfaces are well known, there is no reported example of non-covalent immobilization of adamantane modified NHC with Cucurbit[7]uril (CB7) substrate. Cucurbiturils are a class of macrocyclic molecules with rigid skeletons, carbonyl-laced portals and hydrophobic cavities and are the ideal host for varieties of neutral and cationic species [15]. Among the different classes of cucurbiturils, CB7, in particular, has a superior binding affinity towards adamantane moieties. Hence, in this study, CB7 was chosen in order to immobilize the NHC ligand for a proof-of-principle experiment. Furthermore, the NHC was designed featuring a carboxylate chelating group to provide resistance against reductive elimination under strong reducing conditions [16]. Herein, we report the synthesis, structural analysis and the host–guest interactions of the first adamantane modified NHC on CB7 substrates.

## 2. Experimental Section

### 2.1. General Synthetic Considerations

1H-benzo[d]imidazole-5,6-dicarboxylic acid (**2**) and dimethyl 1H-benzo[d]imidazole-5,6-dicarboxylate (**3**) were prepared as previously described [17,18]. All other materials were of reagent quality and used as received. All solvents used were HPLC grade. ^1^H and ^13^C{^1^H} NMR spectra were recorded using a Bruker 500 MHz spectrometer. Chemical shifts δ (in ppm) for ^1^H and ^13^C NMR are referenced to SiMe_4_ using the residual protio-solvent as an internal standard. For ^1^H NMR: CDCl_3_, 7.26 ppm; DMSO-*d*_6_, 2.50 ppm, CD_3_OD, 3.31 ppm. For ^13^C NMR: CDCl_3_, 77.16 ppm; DMSO-*d*_6_, 39.52 ppm. Coupling constants (*J*) are expressed in hertz (Hz). Infrared spectra were recorded with 1 cm^–1^ resolution on a Shimadzu IRAffinity-1S spectrometer. Elemental analyses were performed at Atlantic Microlab, Inc. (Norcross, GA, USA). When necessary, reactions were performed under an inert atmosphere under an N_2_ atmosphere using standard Schlenk or glovebox techniques with the exclusion of light. All subsequent manipulations were conducted under ambient conditions using standard techniques without the exclusion of light. When required, solvents were dried and deoxygenated using an Innovative Technologies solvent purification system, and then stored over molecular sieves (3 Å) in a dry box.

#### 2.1.1. Synthesis of 1H-Benzo[d]imidazole-5,6-dicarboxylic acid (**2**)

The synthesis of **2** was previously published [17].

#### 2.1.2. Synthesis of Dimethyl 1H-Benzo[d]imidazole-5,6-dicarboxylate (**3**)

In a 500 mL RBF, **2** (4.0 g, 19.42 mmol) was suspended in 200 mL methanol and 4 mL conc. H_2_SO_4_ was added. The reaction mixture was reflux for 16 h. After the reaction, the solvent was evaporated and the viscous liquid was neutralized with 6M K_2_CO_3_ until precipitate ceased forming. 200 mL DCM was added and extracted. The organic layer was then evaporated to dryness. A white solid (4.03 g, 17.22 mmol, 89%). MP 148.0–151.3 °C. ^1^H NMR (500 MHz, CDCl_3_): δ 10.89 (br S, 1H), 8.22 (S, 1H), 8.03 (s, 2H), 3.93 (S, 6H). ^13^C NMR (300 MHz, CDCl_3_): δ 168.78, 144.18, 138.96, 126.96, 117.19, 52.83. ATR-IR: v 2950 (vw), 2590 (w), 1716 (s), 1623 (w), 1422 (w), 1296 (s),1228 (s), 1148 (m), 1101 (m), 1043 (m), 958 (s), 910 (m), 862 (s), 777 (s), 695 (w), 634 (m) cm^−1^ [18].

#### 2.1.3. Synthesis of Dimethyl 1-p-Tolyl-1H-benzo[d]imidazole-5,6-dicarboxylate (**4**)

In a 500 mL RBF, **3** (4.00 g, 17.10 mmol, 1 eq.) and p-tolyboronic acid (3.34 g, 25.65 mmol, 1.5 eq.) were dissolved in 190 mL distilled methanol. To this solution, Cu(NO_3_)_2_ (0.824 g, 3.42 mmol, 20 mol%) and TMEDA (0.255 mL, 1.71 mmol, 10 mol%) were added and stirred at room temperature for 16 h under oxygen [19]. A dark blue solution was observed. After the reaction was completed as evident from TLC, the solvent was removed and the residue was dissolved in 100 mL DCM and extracted with water (50 mL 3 times). The crude product was then purified by flash column chromatography (SiO_2_, 90:10 CH_2_Cl_2_/MeOH) to afford a light yellow solid (5.36 g, 16.54 mmol, 96%) MP: 133.8–134.6 °C, Rf = 0.68. ^1^H NMR (500 MHz, CDCl_3_): δ 8.26 (s, 1H), 8.23 (s, 1H), 7.84 (s, 1H), 7.4–7.36 (m, 4H), 3.94 (s, 3H), 3.89 (s, 3H), 2.47 (s, 3H). ^13^C NMR (500 MHz, CDCl_3_): δ 168.55, 168.19, 145.61, 145.00, 139.16, 135.12, 132.74, 130.86, 128.07, 126.71, 124.25, 122.27, 112.06, 52.77, 52.72, 21.20. ATR-IR: v 3062 (vw), 2950 (vw), 1718 (s), 1619 (vw), 1565 (vw), 1514 (m), 1432 (m), 1366 (m), 1299 (s), 1261 (s), 1224 (s), 1186 (s), 1139 (s), 1101 (s), 1035 (s), 968 (w), 902 (m), 817(s), 770 (s), 713 (w). Ana. Calc. for C_18_H_16_N_2_O_4_: C, 66.66; H, 4.97; N, 8.64. Found: C, 66.77; H, 4.88; N, 8.54.

#### 2.1.4. Synthesis of 1-p-Tolyl-1H-isobenzofuro[5,6-d]imidazole-5,7-dione (**6**)

In a 50 mL RBF, **4** (1.0 g, 3.08 mmol) and potassium hydroxide (375 mg, 6.68 mmol, 2.17 eq.) were suspended in 10 mL methanol/water (9:1). The reaction mixture formed a homogenous light brown solution after stirring for 30 min at room temperature. The reaction was continued for 16 h and then the solvent was evaporated. The viscous liquid was dissolved in minimum water and neutralized with 6M HCl to form a white precipitate. The precipitate was filtered and dried affording a white powder of 1-p-tolyl-1H-benzo[d]imidazole-5,6-dicarboxylic acid **5** (793 mg, 2.68 mmol, 87%). Without further purification and characterization, 1-p-tolyl-1H-benzo[d]imidazole-5,6-dicarboxylic acid **5** was used directly due to poor solubility.

In a 50 mL RBF equipped with condenser, **5** (834 mg, 2.82 mmol) and 9 mL acetic anhydride were reflux for 2 h. The suspension formed homogenous solution after 10 min of refluxing. After the reaction was completed, the light brown solution was cooled to room temperature and then in ice bath to formed precipitate. The reaction mixture was filtered and the precipitate washed with diethyl ether to afford a beige power of 1-p-tolyl-1H-isobenzofuro[5,6-d]imidazole-5,7-dione **6** (667 mg, 2.40 mmol, 85%). mp: 257.5–259.1 °C. ^1^H NMR (500 MHz, CDCl_3_): δ 8.46 (s, 1H), 8.39 (s, 1H), 8.08 (s, 1H), 7.43 (dd, *J* = 30.8 Hz, 4H), 2.51 (s, 3H). ^13^C NMR (500 MHz, CDCl_3_): δ 163.31, 163.10, 149.16, 147.62, 140.22, 139.11, 131.91, 131.16, 126.06, 125.29, 124.42, 119.20, 109.16, 21.26. ATR-IR: v 3101 (vw), 2920 (vw), 2863 (vw), 1836 (m), 1779 (s), 1602 (vw), 1516 (w), 1389 (vw), 1322 (m), 1303 (m), 1265 (m), 1217 (w), 1160 (w), 1068 (vw), 888 (s), 802 (w), 735 (s), 627 (m) cm^−1^. Ana. Calcd. for C_16_H_10_N_2_O_3_: C, 69.06; H, 3.62; N, 10.07. Found: C, 69.00; H, 3.74; N, 10.02.

#### 2.1.5. Synthesis of 6-Adamantanemethyl-1-p-tolylimidazo [4,5-isoindole-5,7(1H,6H)-dione (**7**)

Beige power of **6** (0.95 g, 3.4 mmol) and Adamantanemethylamine (0.56 g, 3.4 mmol, 1 eq.) were placed in 250 mL RBF under N_2_. Then, 50 mL acetic acid was added and reflux for 24 h. After completion of the reaction, the mixture was cooled down to RT and 50 mL distilled water was added to form white precipitate. The precipitate was filtered and washed with water [20]. The white solid obtained was recrystallized in methanol and dried to afford a white powder (1.30 g, 3.06 mmol, 90%) mp: 258.0–259.1 °C. ^1^H NMR (500 MHz, CDCl_3_): δ 8.30 (s, 1H), 8.26 (s, 1H), 7.40 (dd, *J* = 16, 8.5 Hz. 4H), 3.41 (s, 2H), 2.49 (s, 3H), 1.96 (s, 3H), 1.68–1.58 (m, 12H). ^13^C NMR (500 MHz, CDCl_3_): δ 169.13, 169.10, 147.68, 145.61, 139.54, 137.54, 132.51, 130.93, 127.70, 126.79, 124.32, 116.78, 106.98, 49.84, 40.86, 36.75, 35.62, 28.30, 21.21. ATR-IR: v 3082 (vw), 2906 (w), 2847 (vw), 1765 (w), 1706 (s), 1618 (vw), 1519 (m), 1392 (s), 1353 (m), 1210 (w), 1092 (w), 876 (w), 827 (m), 748 (s), 620 (m) cm^−1^. Ana. Calcd. for C_27_H_27_N_3_O_2_: C, 76.21; H, 6.40; N, 9.87 Found: C, 75.97; H, 6.33; N, 9.78.

#### 2.1.6. Synthesis of **8**

Bromoacetic acid (409 mg, 0.96 mmol) and **7** (200 mg, 1.44 mmol) were dissolved with 15 mL of dried toluene in a 100 mL RBF equipped with condenser. The reaction mixture was reflux for overnight. After 16 h, a white precipitate had formed. The reaction mixture was allowed to cool to room temperature and the precipitate was collected by centrifugation. The resulting solid was washed with 15 mL toluene (3 times) and was then dried in vacuo to afford a white powder (519 mg, 0.922 mmol, 95% yield). Mp: > 260 °C. ^1^H NMR (500 MHz, CD_3_OD): δ 10.21 (s, 1H),8.4 (d, 0.5Hz, 1H), 8.22 (d, 1Hz, 1H), 7.77 (d, 8.5 Hz, 2H), 7.64 (d, 8 Hz, 2H), 5.71 (s, 2H), 3.44 (S, 2H), 2.57 (s, 3H), 1.97 (s, 3H), 1.77–161 (m, 12H). ^13^C NMR (300 MHz, CD_3_OD): δ167.20, 167.11, 167.08, 146.14, 142.21, 135.59, 135.01, 131.02,130.84, 130.73,130.05, 124.89, 110.26, 109.45, 49.94, 40.61, 36.39, 35.19, 28.36, 19.94. ATR-IR: v 3159 (vw), 2893 (w), 2841 (vw), 1748 (w), 1709 (s), 1629 (vw), 1553 (w), 1430 (w), 1383 (m), 1343 (w), 1257 (vw), 1190 (m), 1169 (s), 1083 (m), 984 (vw), 830 (vw), 744 (m), 618 (s) cm^−1^. Ana. Calcd. for C_29_H_30_BrN_3_O_4_.0.3H_2_O: C, 61.12; H, 5.41; N, 7.37. Found: C, 60.91; H, 5.28, N, 7.12.

### 2.2. Crystal Structure Data Acquisition

A specimen of **8**, approximate dimensions 0.051 mm × 0.202 mm × 0.265 mm, was used for the X-ray crystallographic analysis. The X-ray intensity data were measured. The integration of the data using an orthorhombic unit cell yielded a total of 38,548 reflections to a maximum θ angle of 25.50° (0.83 Å resolution), of which 5780 were independent (average redundancy 6.669, completeness = 99.6%, R_int_ = 8.39%, R_sig_ = 5.16%) and 4770 (82.53%) were greater than 2σ(F^2^). The final cell constants of a = 12.5299(12) Å, b = 49.749(5) Å, c = 10.0582(9) Å, volume = 6269.8(10) Å^3^, are based upon the refinement of the XYZ-centroids of reflections above 20 σ(I). The calculated minimum and maximum transmission coefficients (based on crystal size) are 0.7241 and 1.0000. The structure was solved and refined using the Bruker SHELXTL Software Package, using the space group I b a 2, with Z = 8 for the formula unit, C_31.50_H_36_BrN_3_O_4_.

## 3. Results and Discussion

### 3.1. Design and Synthesis

The synthesis of **8** took into consideration of key features for prospective transition metal catalysts. First, adamantane moiety was strategically incorporated into the NHC structure to serve as a point of supramolecular attachment to solid supports for recyclable homogenous catalysts. Second, a carboxylate chelating group which is reported to afford incredible resistance against reductive elimination under strong reducing conditions was introduced [16]. Our synthesis began with the oxidation of the commercially available precursor 5,6-dimethylbenzimidazole **1** to yield compound **2** according to reported precedures [17]. **2** was subsequently converted into an ester according to Shelton et al. albeit slightly modified procedure to form compound **3** with higher yield and the NMR and MP data conforms with reported values [18]. This modification allows **3** to be soluble in various organic solvents which was not possible for **2**. The synthesis of compound **4**–**8** has never been reported.

Using a mild reaction condition involving Cu(NO_3_)_2_-TMEDA complex in methanol at room temperature, compound **3** was coupled with p-Tolylboronic acid to render 96% yield of compound **4** following a straight forward flash chromatography in 1% methanol in dichloromethane.

**Scheme 1:**
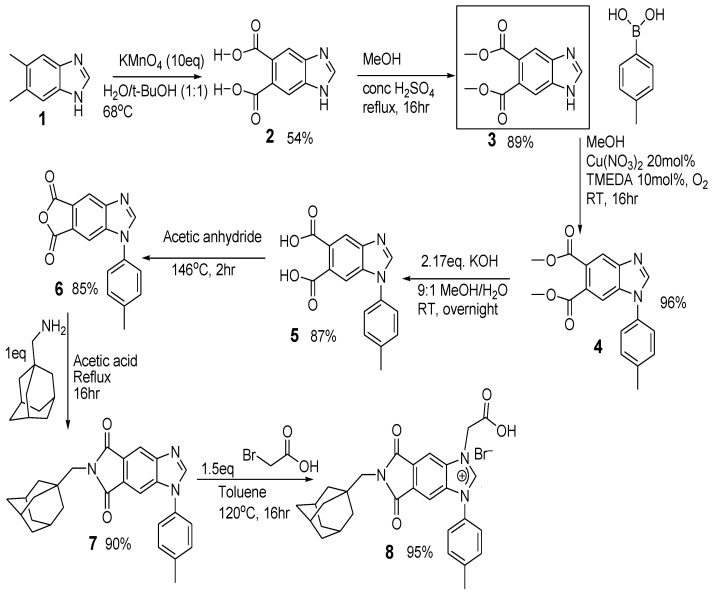
Preparation of **8** with 5,6-dimethylbenzimidazole as the starting material.

**Figure 1:**
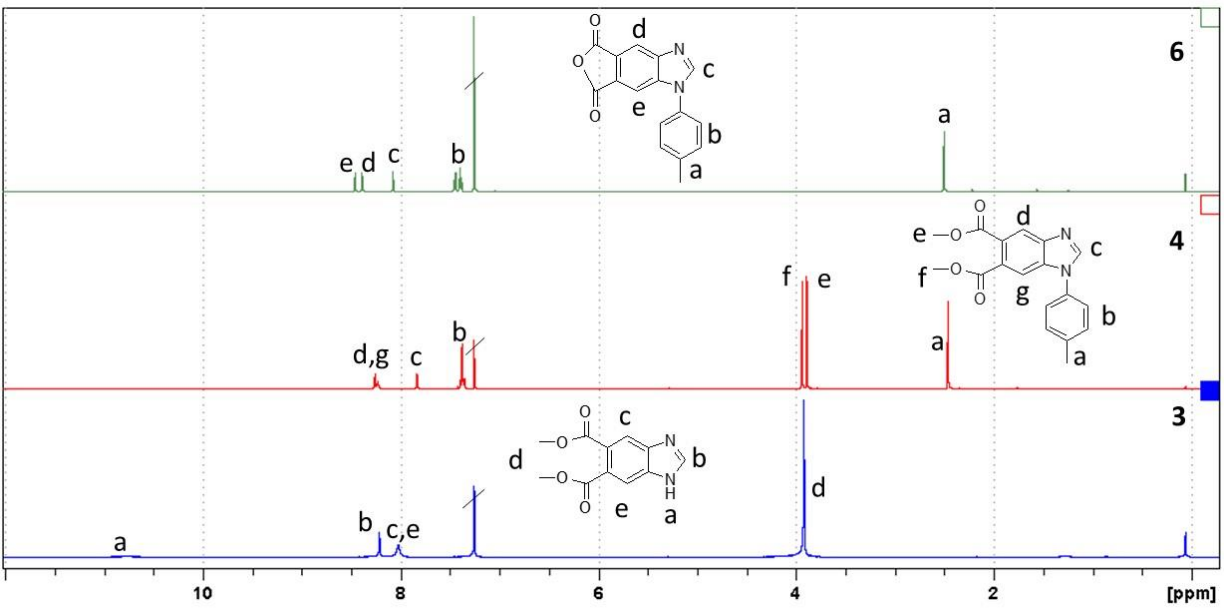
Overlay of ^1^H-NMR of compound **3**, **4** and **6** in CDCl_3_.

To obtain the anhydride **6** (Scheme 1), compound **4** had to be hydrolyzed into the corresponding dicarboxylic acid **5** under basic conditions. Without further purification, due to the poor solubility of **5**, dried solid of **5** was refluxed in acetic anhydride to render the desired product **6.** The ^1^H NMR spectrum indicates a successful synthesis of **6** and in comparison to ^1^H NMR of **4**, no peaks at 3.94 ppm and 3.90 ppm (δ CDCl_3_) were observed (Figure 1). This confirms the successful hydrolysis of methyl ester **4** and the subsequent formation of an anhydride **6**. The ^1^H NMR spectrum of **4** (Figure 1) showed a singlet at 2.47 ppm and doublet of doublet at 7.42–7.36 ppm (δ CDCl_3_) belonging to the methyl protons and aromatic protons of p-tolyl group respectively. Due to the asymmetrical nature of compound **4**, the chemical shift of the two methyl esters at the 5,6 position of the benzimidazole differs slightly compare to starting material **3** where only a singlet was observed at 3.93 ppm.

**Figure 2:**
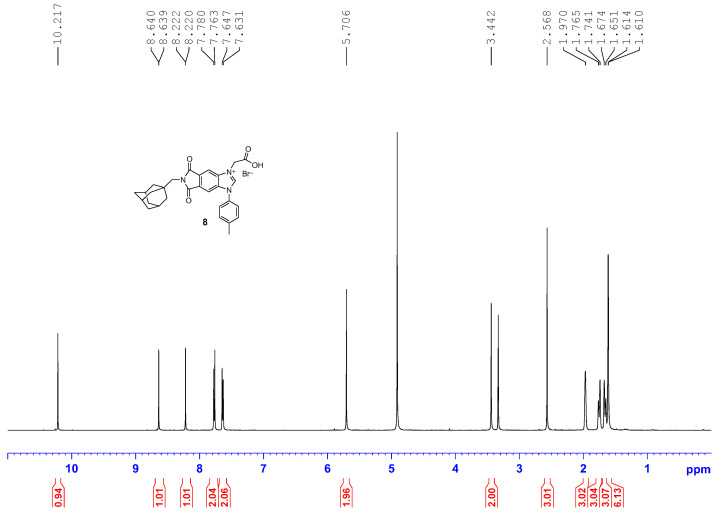
^1^H-NMR (CDCl3) spectrum of **8**.

The phthalic anhydride moiety in **6** is an excellent precursor for attaching adamantyl group for supramolecular capabilities with host molecules. By appending an adamantyl group at this position, we postulate that it will have a minimal electronic or steric effect on the metal center of the NHC. Accordingly, **7** was synthesized by refluxing **6** and 1-Adamantanemethylamine in acetic acid for 16h. ^1^H NMR spectrum of **7** (see Supplementary Materials) showed methylene protons at 3.4 ppm and adamantane protons ranging from 1.95 ppm to 1.57 ppm (δ CDCl_3_). Previous attempts to functionalized **6** with 1-Adamantaneamine were unsuccessful presumably due to steric reasons. A mixture of mono-substituted, starting material and product were observed, each with very close Rf values. As a result, 1-Adamantanemethylamine was selected for this project since it is less sterically demanding than 1-Adamantaneamine due to the presence of a methylene group. With a pure sample of **7** at our disposal, the benzimidazolium salt **8** was synthesized by reacting **7** with 1.5eq bromoacetic acid in toluene at 120 °C for 16 h. Slowly, a white solid began to form in toluene as the insoluble benzimidazolium salt forms. Benzimidazolium salt **8** was easily filtered out and washed with toluene to obtain a white solid with a 95% yield. The ^1^H NMR shows a significant chemical shift of compound **7** proton at the C1-position from 7.93 ppm to 10.17 ppm (δ CD_3_OD) upon functionalization with bromoacetic acid to form **8** (Figure 2). Compound **8** was designed to coordinate with transition metals through the carbene carbon and oxygen atom from the carboxylate substituent which could offer a chelating effect. This property makes **8** a desirable precursor for the synthesis of *N*-Heterocyclic carbene of silver, ruthenium and other metal complexes [21,22,23].

### 3.2. Host-Guest Complexation of ***8*** and CB7

**Scheme 2:**
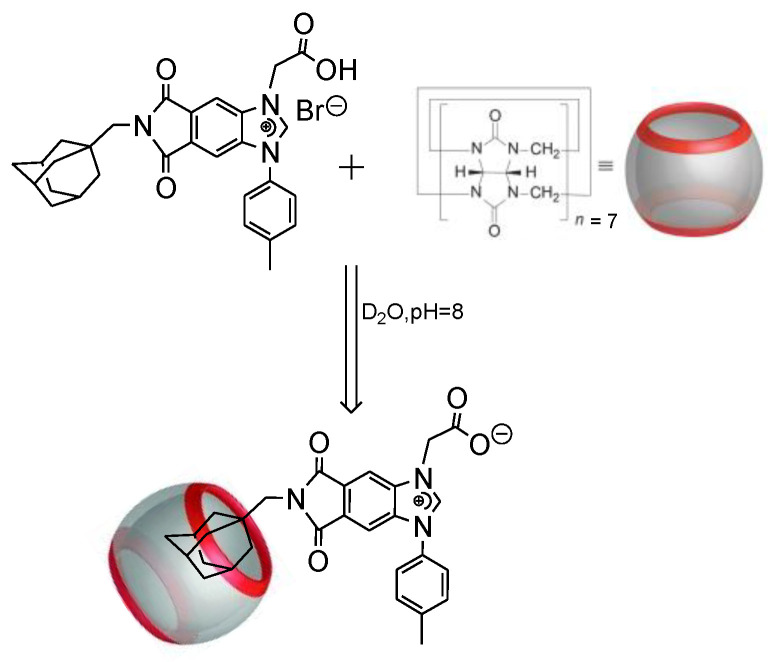
Host-guest inclusion complex of NHC **8** and CB7.

To demonstrate host–guest interactions between adamantyl moiety and CB7, we chose **8** and titrated it against increasing concentration of CB7 in deuterated water at pH = 8 (Scheme 2). Accordingly, 0.2, 0.4, 0.6, 0.8, 1.0, 1.2 equivalent CB7 (in D_2_O) was added sequentially to **8** and their chemical shifts were recorded with reference to the ^1^HNMR of **8** only.

**Figure 3:**
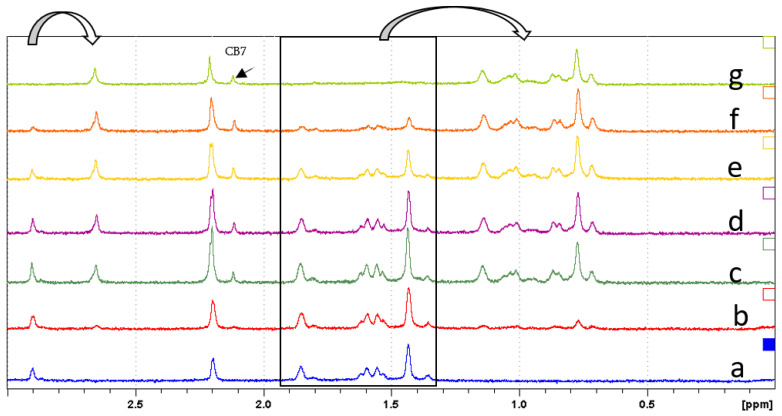
Partial NMR spectra (300 MHz, D_2_O) of **8** (2.23 mM) containing: (a) no CB7, (b) 0.2eq CB7(20 μL), (c) 0.4 eq. CB7, (d) 0.6 eq. CB7, (e) 0.8 eq. CB7, (f) 1.0 eq. CB7, (g) 1.2 eq. CB7. The black rectangle region represents the free adamantyl protons.

In Figure 3, upon adding 0.2 eq. of CB7 to **8**, the adamantyl protons shifted upfield from 1.30–1.80 ppm to 0.60–1.20 ppm as indicated by the arrow. As an incremental amount of CB7 was added, the peaks in the rectangle region were shifted to the right indicating the formation of host–guest inclusion complex. After adding 1.2 eq. CB7, the peaks from 1.30–1.80 ppm shifted completely which corresponds to the difference in chemical environment between the free and encapsulated adamantyl moiety. The methylene group linking the adamantyl to the benzimidazolium moiety (Scheme 2) is also sensitive to CB7 inclusion shifting from 2.90 ppm to 2.66 ppm. Accordingly, the binding constant was determined from ^1^H NMR titration experiment to be K_a_ = 9.5 × 10^8^ M^−1^. The results clearly showed that **8** can form a stable 1:1 inclusion complex with CB7. This further reinforces our hypothesis that 8 could be immobilized on solid supports that are functionalized with CB7 via non-covalent interactions.

### 3.3. Solid State Analysis

**Figure 4:**
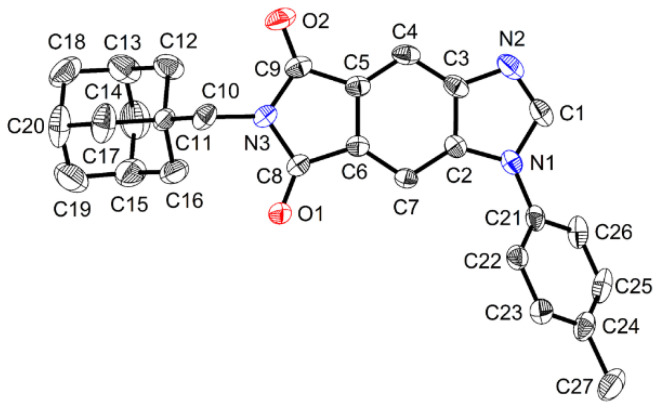
ORTEP diagram of compound **7**. Thermal ellipsoids are shown at the 50% probability level. Hydrogen atoms have been omitted for the purpose of clarity. Selected distances (Å) and angles (^ο^): O1-C8, 1.212(2); O2-C9, 1.210(2); N1-C1, 1.370(2); N1-C2, 1.387(2); N1-C21, 1.429(2); N2-C1, 1.306(3), N2-C3, 1.386(2); N3-C8, 1.399(2); N3-C9, 1.400(2); N3-C10, 1.460(2). C1-N1-C2, 106.03(16); C1-N1-C21, 125.60(16); C2-N1-C21, 128.34(15); C1-N2-C3, 104.52(16); C8-N3-C9, 111.51(15); C8-N3-C10, 123.37(15); C9-N3-C10, 124.47(15); N2-C1-N1, 114.37(17).

Representative crystals were analyzed by single-crystal X-ray diffraction. A single crystal of **7** was obtained from chloroform solution by slow vapor deposition of ether at room temperature. Crystallographic analysis indicated a monoclinic crystal system and P 1 21/c 1 space group. Since carbene carbon of NHCs plays a critical role as the donor atom in the formation of metal-NHC complex, bond lengths of 1.370(2) (Å) and 1.306(3) (Å) were, respectively, determined for C1-N1 and C1-N2 (Figure 4). The N3-C10-C11 bond angle is 115.81(15) (^ο^). The crystal structure clearly showed that the adamantyl moiety is anchored furthest from the C1 position thereby avoiding potential steric hindrance to prospective metal complex formation.

**Figure 5:**
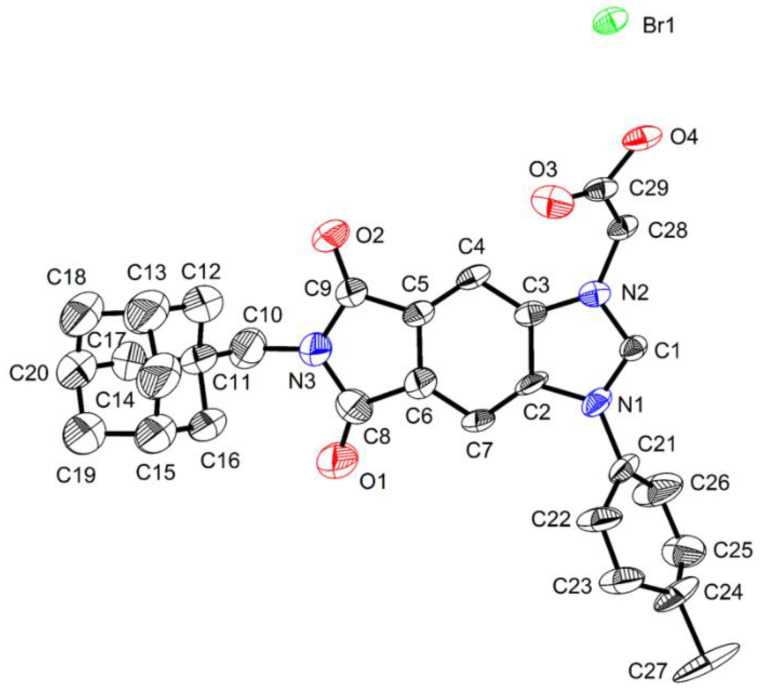
ORTEP diagram of compound (**8**). Thermal ellipsoids are shown at the 50% probability level. Hydrogen atoms have been omitted for the purpose of clarity. Selected distances (Å) and angles (^ο^): O1-C8, 1.208(15); O2-C9, 1.234(12); O3-C29, 1.212(13); O4-C29, 1.355(12); N1-C1, 1.341(11), N1-C2, 1.397(12); N1-C21, 1.464(11); N2-C1, 1.359(11); N2-C3, 1.376(12); N2-C28, 1.467(12); N3-C9, 1.395(14); N3-C8, 1.411(17); N3-C10, 1.479(18). C29-O4-H4, 109.5; C1-N1-C2, 108.4(7); C1-N1-C21, 123.7(7); C2-N1-C21, 127.5(7); C1-N2-C3, 108.3(7); C1-N2-C28, 121.4(8); C3-N2-C28, 129.8(8); C9-N3-C8, 112.5(9); C9-N3-C10, 123.7(11); C8-N3-C10, 123.0(11); N1-C1-N2, 110.0(7).

Single crystals of **8** were obtained from saturated chloroform solution by slow vapor deposition of pentane at room temperature. Crystallographic analysis indicated an orthorhombic crystal system and Iba2 space group. The bond length of N2-C1, 1.359(11) Å in **8** (Figure 5) is longer than its precursor **7**, N2-C1, 1.306(3) Å (Figure 4). A plausible explanation for this is that the carboxylate substituent acts as an electron-withdrawing group and thereby withdraws electron density from the five-membered ring, hence elongating the C1-N2 bond. The bond angle of C1-N2-C28 is 121.4(8) (^ο^), providing a favorable bite angle for bidentate chelation by the C1 and O4 donor groups of the NHC **8** with transition metals.

## 4. Conclusions

A novel NHC was designed and synthesized with non-covalent Immobilization capabilities. Adamantyl moiety was incorporated into the NHC structure as a guest molecule for supramolecular interactions with CB7 substrates. A proof-of-principle non-covalent Immobilization of **8** with CB7 was illustrated using ^1^H-NMR titration. The NMR data showed the adamantyl protons shifted upfield upon formation of inclusion complex with CB7. The result showed a 1:1 NHC-CB7 supramolecular complex formation, hence its potential to be used in surface Immobilizable homogeneous catalysts. The addition of a carboxylate group offered the NHC bidentate chelation features through the oxygen and carbene donor atoms. The structure of the NHC ligand (**8**) was confirmed by ^1^H-NMR, ^13^C-NMR, FTIR, single-crystal X-ray crystallography and elemental analysis.

## Data Availability

The data reported in this study are available in the Supplementary Materials. Crystallographic data can be obtained free of charge via www.ccdc.cam.ac.uk/data_request/cif, access on 24 January 2022, or by emailing data_request@ccdc.cam.ac.uk. CCDC #: 2144304 and CCDC #: 2144302 respectively for compound **7** & **8**.

## Supplementary Materials

The following are available online at. A PDF file of the NMR and FTIR spectra of all reported compounds and crystal structure report for compound **7** & **8**.

## Sample Availability

Samples of the compound **8** is not available from the author.

## Abbreviations

Ad	Adamantane
CB7	Curcubit[7]uril
NHC	*N*-Heterocyclic Carbene
NMR	Nuclear Magnetic Resonances
TMED	Tetramethylethylenediamine
RBF	Round Bottom Flask
