# Supplementary material for: Supramolecular Immobilization of Adamantyl and Carboxylate Modified N-Heterocyclic Carbene Ligand on Cucurbituril Substrates
Source: Molecules. 2022 Mar 3;27(5):1662. doi: 10.3390/molecules27051662 (PMC8911794; doi:10.3390/molecules27051662)
Supplement: Supplementary file 1 [file molecules-27-01662-s001.zip › molecules-1591015 supplementary.pdf]

molecules

Supplementary Materials

Supramolecular Immobilization of Adamantyl and Carboxylate Modified *N*-Heterocyclic Carbene Ligand on Cucurbituril Substrates

Hamidou Keita

Departments of Chemistry, Clemson University, Clemson, SC 29634, USA

1. Experimental section

1.1. General synthetic considerations

1H-benzo[d]imidazole-5,6-dicarboxylic acid (**2**) and dimethyl 1H-benzo[d]imidazole-5,6-dicarboxylate (**3**) were prepared as previously described[28,29]. All other materials were of reagent quality and used as received. All solvents used were HPLC grade. ^{1}H and $^{13}C\{^{1}H\}$ NMR spectra were recorded using a Bruker 500 MHz spectrometer. Chemical shifts δ (in ppm) for ^{1}H and ^{13}C NMR are referenced to $SiMe_4$ using the residual protio-solvent as an internal standard. For ^{1}H NMR: $CDCl_3$, 7.26 ppm; DMSO-d_6, 2.50 ppm, CD_3OD, 3.31ppm. For ^{13}C NMR: $CDCl_3$, 77.16 ppm; DMSO-d_6, 39.52 ppm. Coupling constants (*J*) are expressed in hertz (Hz). Infrared spectra were recorded with 1 cm^{-1} resolution on a Shimadzu IRAffinity-1S spectrometer. Elemental analyses were performed at Atlantic Microlab, Inc. (Norcross, GA). All reactions were performed under an inert atmosphere under an N_2 atmosphere using standard Schlenk or glovebox techniques with the exclusion of light. All subsequent manipulations were performed under ambient conditions using standard benchtop techniques without the exclusion of light. When required, solvents were dried and deoxygenated using an Innovative Technologies solvent purification system, and then stored over molecular sieves (3 Å) in a drybox. For X-ray characterization, the structure was solved and refined using Bruker SHELXTL Software. The ruthenium complex was characterized with UV-VIS spectrophotometer (Shimadzu UV-2600).

Synthesis of 1H-benzo[d]imidazole-5,6-dicarboxylic acid (BDCOOH) (2)

In a 2L conical flask, 5,6-dimethylbenzimidazole (8.00g, 0.0547mol) was added followed by 140ml (1:1 v/v) of water and tert-butanol and the mixture was heated to 70°C for 15min while stirring. To the homogenous solution, $KMnO_4$ (86.40g, 0.547mol) dissolved in 600ml water at 70°C was added in 20ml portions over 4hr period. The temperature of the reaction mixture was maintained at 70°C throughout. After 4hr, the heat was turned off and 30.00g (0.238mol) sodium sulfite was added in 5 portions and the hot mixture was stirred for 30mins and filtered. The black cake was washed with 100ml boiling water. The filtrate was concentrated to 300 mL and diluted to 600ml with distilled water. To the cooled solution,120ml (2:1 v/v) acetic acid and water mixture was added to form white precipitate which was filtered and dried. A white solid (6.06g,29.4mmol, 54%). ^{1}H NMR (DMSO-d_6): δ 12.64 (s, 1H), 8.51(s, 1H), 7.90 (s, 1H). ^{13}C NMR (DMSO-d6): δ 169.5, 145.7, 138.4, 128.2, 116.5.[28]

Synthesis of dimethyl 1H-benzo[d]imidazole-5,6-dicarboxylate (BDCOAc) (3)

In a 500ml RBF, **2** (4.0g, 19.42mmol) was suspended in 200ml methanol and 4ml conc. H_2SO_4 was added. The reaction mixture was reflux for 16hr. After the reaction, the solvent was evaporated and the viscous liquid was neutralized with 6M K_2CO_3 until precipitate

ceased forming. 200ml DCM was added and extracted. The organic layer was then evaporated to dryness. A white solid (4.03g, 17.22mmol, 89%). MP 148.0-151.3°C. ^{1}H NMR (500MHz, $CDCl_3$): δ 10.89 (br S, 1H), 8.22 (S, 1H), 8.03 (s, 2H), 3.93 (S, 6H). ^{13}C NMR (300MHz, $CDCl_3$): δ 168.78, 144.18, 138.96, 126.96, 117.19, 52.83. ATR-IR : v 2950 (vw), 2590 (w), 1716 (s), 1623 (w), 1422 (w), 1296 (s),1228 (s), 1148 (m), 1101 (m), 1043 (m),958 (s), 910 (m), 862 (s), 777 (s), 695 (w), 634 (m) cm^{-1}. [29]

Synthesis of dimethyl 1-p-tolyl-1H-benzo[d]imidazole-5,6-dicarboxylate (BTolOAc) (4)[30]

In a 500ml RBF, **3** (4.00g, 17.10mmol,1eq) and p-tolyboronic acid (3.34g, 25.65mmol,1.5eq) were dissolved in 190ml distilled methanol. To this solution, $Cu(NO_3)_2$ (0.824g, 3.42mmol, 20m0l%) and TMEDA (0.255mL, 1.71mmol, 10mol%) were added and stirred at room temperature for 16hr under oxygen. A dark blue solution was observed. After the reaction was completed as evident from TLC, the solvent was removed and the residue was dissolved in 100mL DCM and extracted with water (50mL x3). The crude product was then purified by flash column chromatography (SiO_2, 90:10 CH_2Cl_2/MeOH) to afford a light yellow solid (5.36g, 16.54mmol, 96%) MP: 133.8-134.6^{0}C, Rf = 0.68. ^{1}H NMR (500MHz, $CDCl_3$): δ 8.26 (s, 1H), 8.23 (s,1H), 7.84 (s,1H), 7.4-7.36 (m, 4H), 3.94 (s,3H), 3.89 (s,3H), 2.47 (s, 3H). ^{13}C NMR (500MHz, $CDCl_3$): δ 168.55, 168.19,145.61,145.00,139.16, 135.12, 132.74, 130.86, 128.07, 126.71, 124.25, 122.27,112.06,52.77, 52.72, 21.20. ATR-IR: v 3062(vw), 2950(vw), 1718(s), 1619(vw), 1565(vw), 1514(m), 1432(m), 1366(m), 1299 (s), 1261(s), 1224(s), 1186(s), 1139(s), 1101(s), 1035(s), 968(w), 902 (m), 817(s), 770(s), 713(w). Ana. Calc. for $C_{18}H_{16}N_2O_4$: C, 66.66; H,4.97; N, 8.64. Found: C, 66.77; H,4.88; N, 8.54.

Synthesis of 1-p-tolyl-1H-isobenzofuro[5,6-d]imidazole-5,7-dione (BTolAnh)(6)

In a 50ml RBF, **4** (1.0g, 3.08mmol) and potassium hydroxide (375mg, 6.68mmol, 2.17eq) were suspended in 10ml Methanol/water (9:1). The reaction mixture formed homogenous light brown solution after stirring for 30mins at RT. The reaction was continued for 16h and then the solvent was evaporated. The viscous liquid was dissolved in minimum water and neutralized with 6M HCl to formed a white precipitate. The precipitate was filtered and dried affording a white powder of 1-p-tolyl-1H-benzo[d]imidazole-5,6-dicarboxylic acid **5** (793mg,2.68mmol,87%). Without further purification, 1-p-tolyl-1H-benzo[d]imidazole-5,6-dicarboxylic acid **5** was used directly.

In a 50ml RBF equipped with condenser, **5** (834mg, 2.82mmol) and 9ml acetic anhydride were reflux for 2hr.The suspension formed homogenous solution after 10min of refluxing. After the reaction was completed, the light brown solution was cooled to room temperature and then in ice bath to formed precipitate. The reaction mixture was filtered and the precipitate washed with diethyl ether to afford a beige power of 1-p-tolyl-1H-isobenzofuro[5,6-d]imidazole-5,7-dione **6** (667mg, 2.40mmol, 85%). mp: 257.5-259.1°C. ^{1}H NMR (500MHz, $CDCl_3$): δ 8.46 (s, 1H), 8.39 (s, 1H), 8.08 (s, 1H), 7.43 (dd, J=30,8Hz, 4H), 2.51 (s, 3H). ^{13}C NMR (500MHz, $CDCl_3$):δ 163.31, 163.10, 149.16, 147.62, 140.22, 139.11, 131.91, 131.16, 126.06, 125.29, 124.42, 119.20, 109.16, 21.26. ATR-IR: v 3101 (vw), 2920 (vw), 2863 (vw), 1836 (m), 1779 (s), 1602 (vw), 1516 (w), 1389 (vw), 1322 (m), 1303 (m), 1265 (m), 1217 (w), 1160 (w), 1068 (vw), 888 (s), 802 (w), 735 (s), 627 (m) cm^{-1}. Ana. Calcd. for $C_{16}H_{10}N_2O_3$: C, 69.06; H, 3.62; N, 10.07. Found: C, 69.00; H, 3.74; N, 10.02.

Synthesis of 6-adamantanemethyl-1-p-tolylimidazo [4,5-f]isoindole-5,7(1H,6H)-dione (BTadme) (7)

Beige power of **6** (0.95g, 3.4mmol) and adamantanemethylamine (0.56g, 3.4mmol, 1eq.) were placed in 250mL RBF under N_2. Then 50mL acetic acid was added and reflux for 24hr. After completion of the reaction, the mixture was cooled down to RT and 50mL distilled water was added to form white precipitate. The precipitate was filtered and washed with water. The white solid obtained was recrystallized in methanol and dried to afford a white powder (1.30g, 3.06mmol, 90%) mp: 258.0-259.1^{0}C. ^{1}H NMR (500MHz, $CDCl_3$): δ 8.30 (s, 1H), 8.26 (s, 1H), 7.40 (dd, J=16, 8.5Hz. 4H), 3.41 (s, 2H), 2.49 (s, 3H), 1.96 (s, 3H), 1.68-1.58 (m, 12H). ^{13}C NMR (500MHz, $CDCl_3$): δ 169.13, 169.10, 147.68, 145.61, 139.54, 137.54, 132.51, 130.93, 127.70, 126.79, 124.32, 116.78, 106.98, 49.84, 40.86, 36.75, 35.62, 28.30, 21.21. ATR-IR: v 3082 (vw), 2906 (w), 2847 (vw), 1765 (w), 1706 (s), 1618 (vw), 1519

(m), 1392 (s), 1353 (m), 1210 (w), 1092 (w), 876 (w), 827 (m), 748 (s), 620 (m) cm^{-1}. Ana. Calcd. for $C_{27}H_{27}N_3O_2$: C,76.21; H, 6.40; N, 9.87   Found: C, 75.97; H, 6.33; N, 9.78

Synthesis of BToladBr (8)

Bromoacetic acid (409 mg, 0.96 mmol) and **7** (200mg, 1.44mmol) were dissolved with 15 mL of dried toluene in a 100 mL RBF equipped with condenser. The reaction mixture was reflux for overnight. After 16h, a white precipitate had formed. The reaction mixture was allowed to cool to room temperature and the precipitate was collected by centrifugation. The resulting solid was washed with 15 mL x 3 toluene and was then dried in vacuo to afford a white powder (519 mg, 0.922mmol, 95% yield). Mp: > 260°C. ^{1}H NMR (500MHz, CD_3OD): δ 10.21 (s, 1H),8.4 (d, 0.5Hz, 1H), 8.22 (d, 1Hz, 1H), 7.77 (d, 8.5Hz, 2H), 7.64 (d, 8Hz, 2H), 5.71 (s, 2H), 3.44 (S, 2H), 2.57 (s, 3H), 1.97 (s, 3H), 1.77-161 (m, 12H). ^{13}C NMR (300MHz, CD_3OD): δ167.20, 167.11, 167.08, 146.14, 142.21, 135.59, 135.01, 131.02,130.84, 130.73,130.05, 124.89, 110.26, 109.45, 49.94, 40.61, 36.39, 35.19, 28.36, 19.94. ATR-IR: ν 3159 (vw), 2893 (w), 2841 (vw), 1748 (w), 1709 (s), 1629 (vw), 1553 (w), 1430 (w), 1383 (m), 1343 (w), 1257 (vw), 1190 (m), 1169 (s), 1083 (m), 984 (vw), 830 (vw), 744 (m), 618 (s) cm^{-1}. Ana. Calcd. for $C_{29}H_{30}BrN_3O_4.0.3H_2O$: C, 61.12; H, 5.41; N, 7.37. Found: C, 60.91; H, 5.28, N, 7.12.

FTIR spectrum of **2**

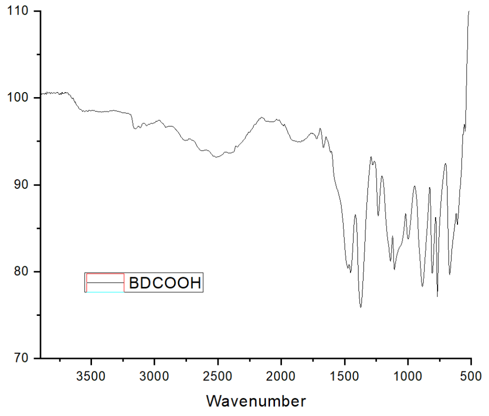

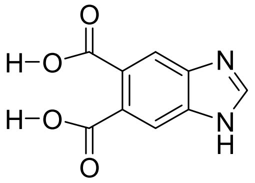

2

FTIR spectrum of **3**

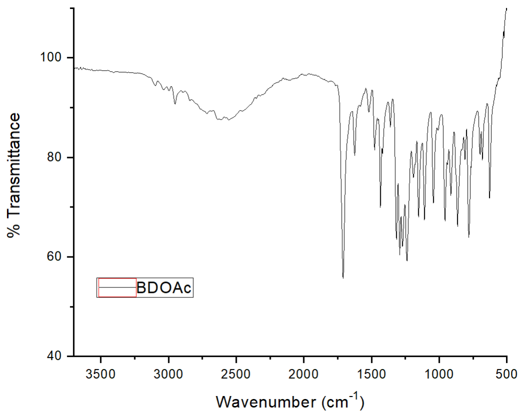

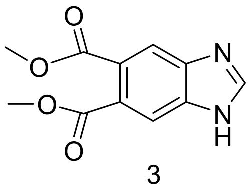

3

FTIR spectrum of **4**

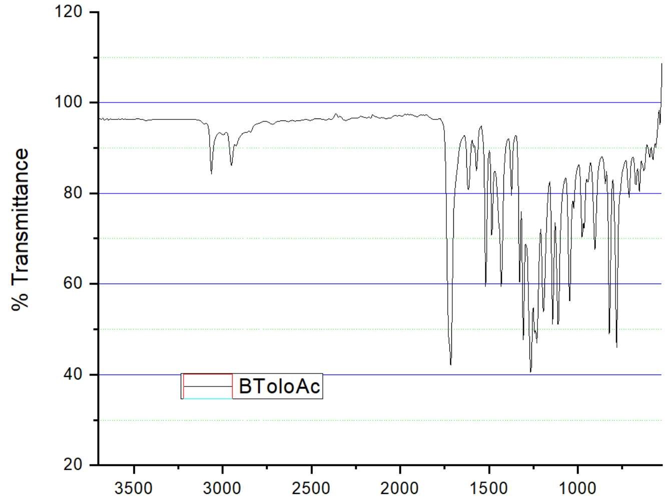

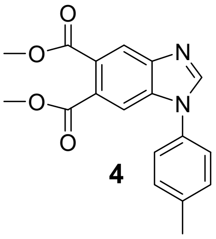

4

FTIR spectrum of **6**

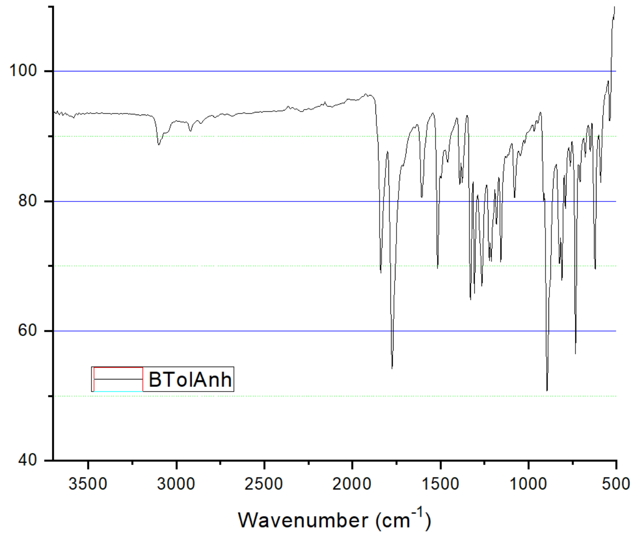

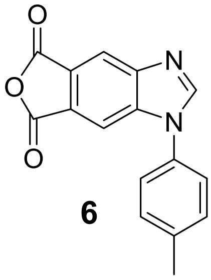

6

FTIR spectrum of **7**

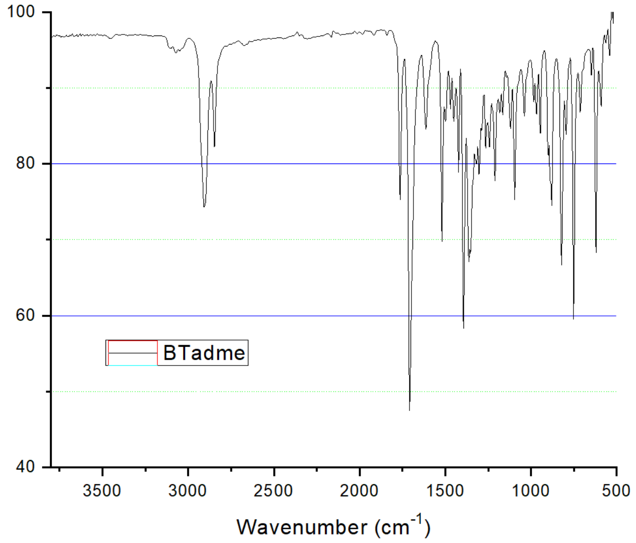

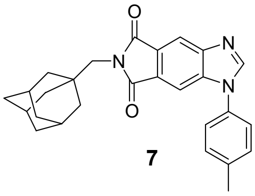

7

FTIR spectrum of **8**

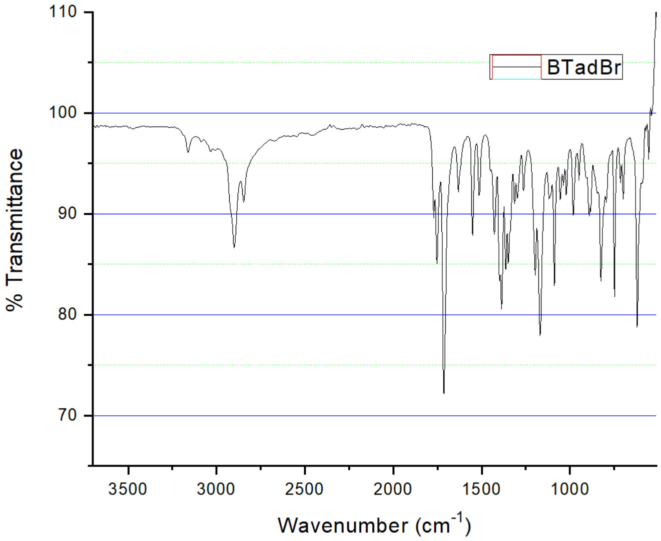

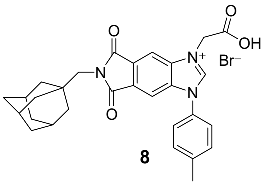

8

^{1}H-NMR (DMSO-d$_6$) spectrum of **2**

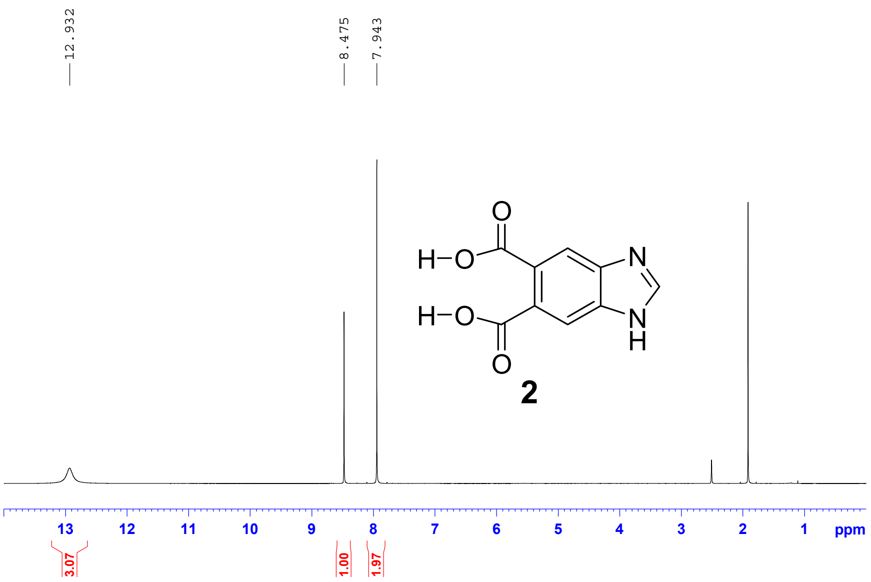

^{13}C-NMR (DMSO-d$_6$) spectrum of **2**

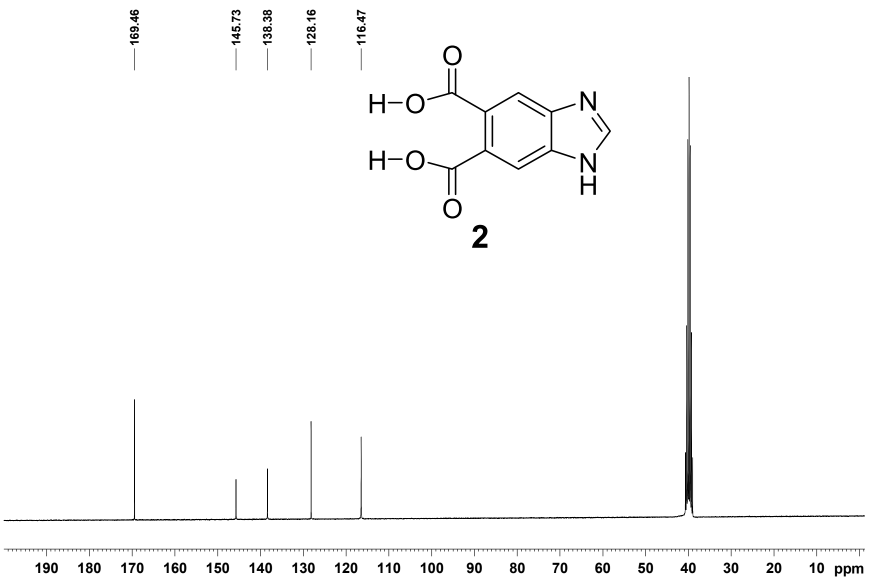

^{1}H-NMR (CDCl3) spectrum of **3**

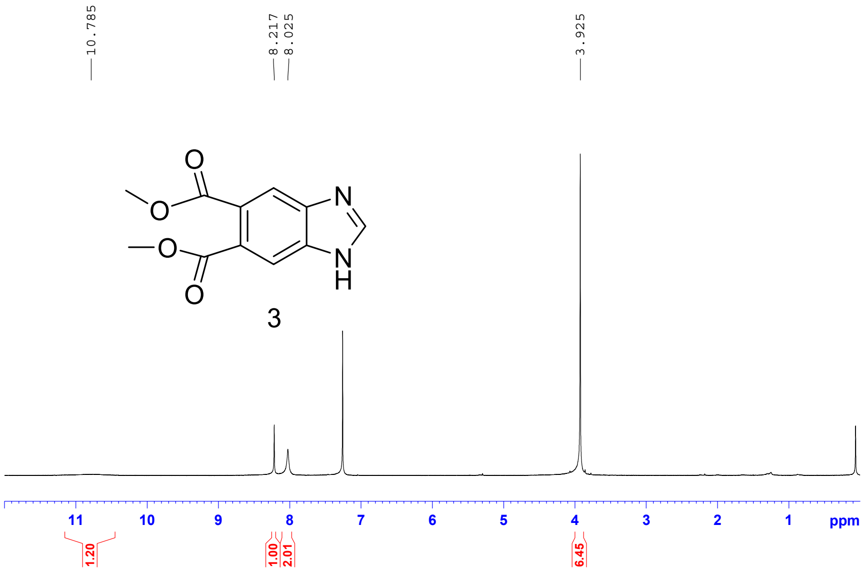

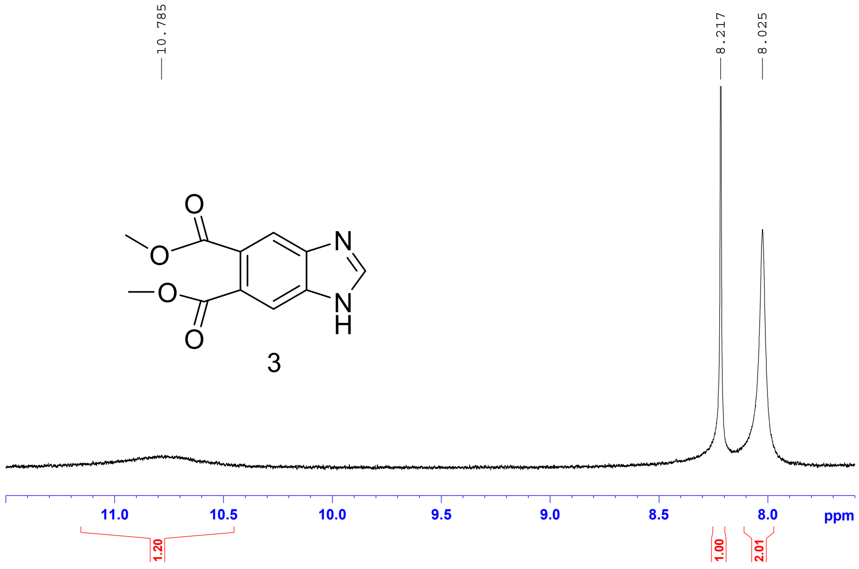

^{13}C-NMR (CDCl3) spectrum of **3**

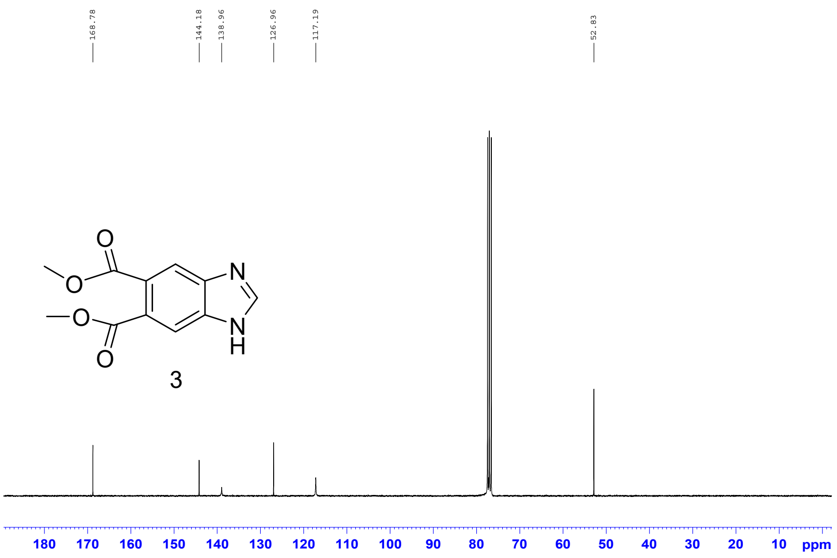

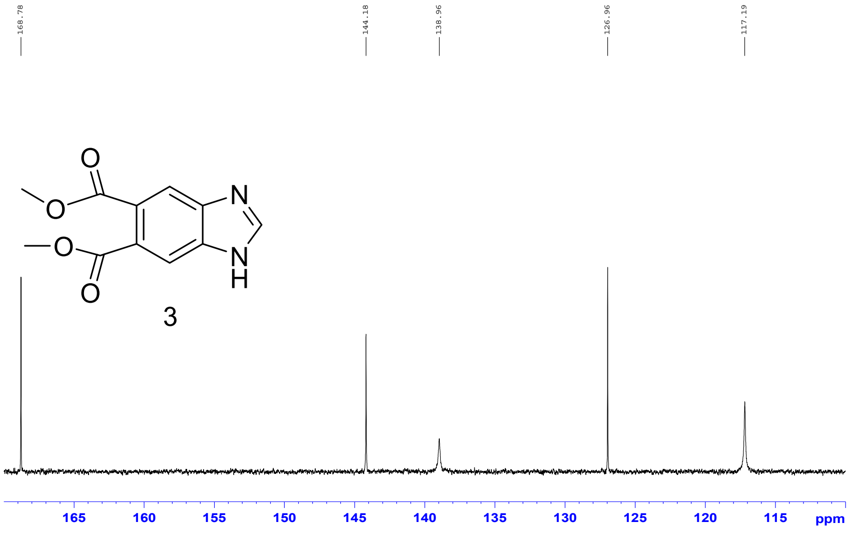

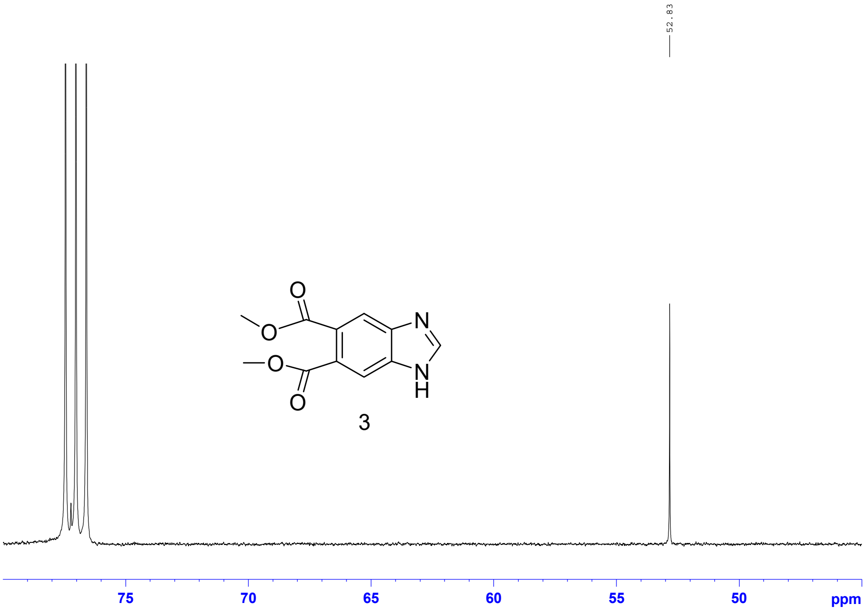

^{1}H-NMR (CDCl3) spectrum of **4**

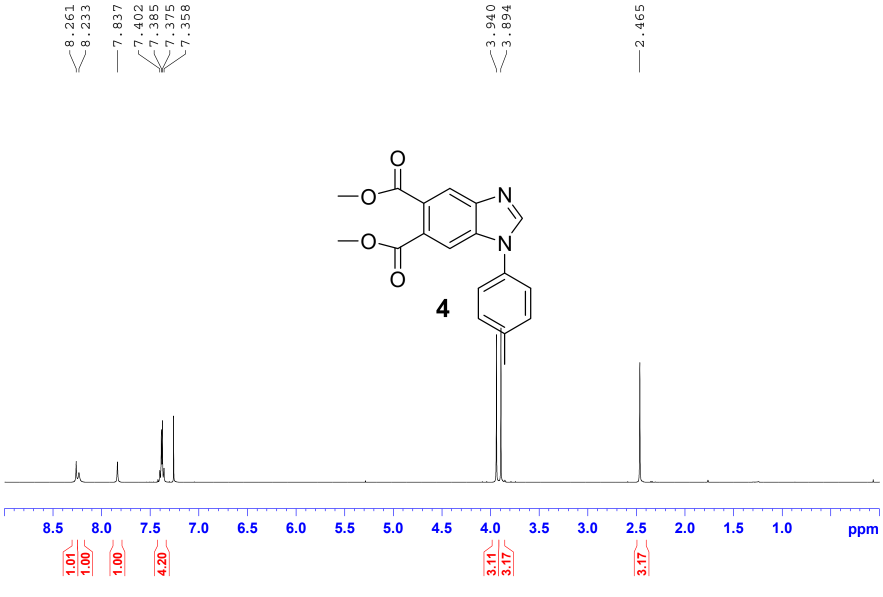

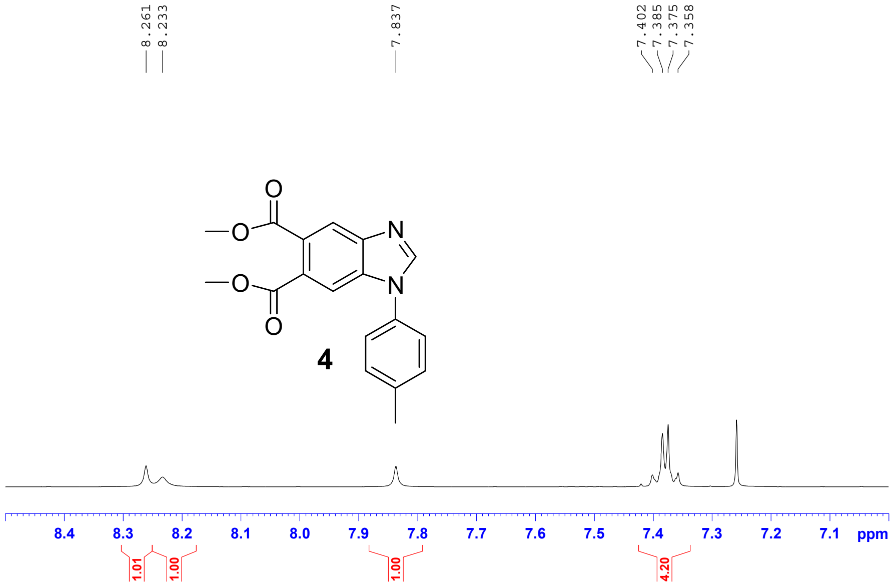

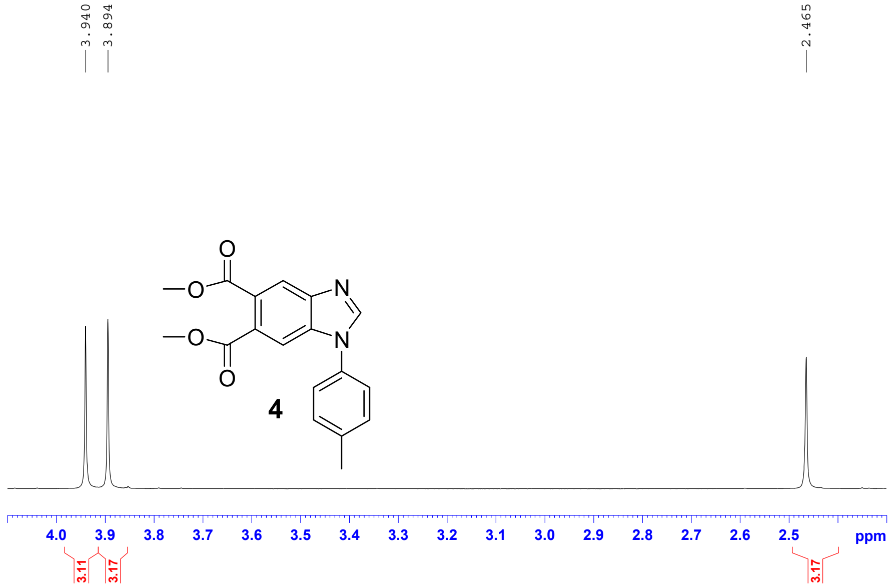

^{13}C-NMR (CDCl3) spectrum of **4**

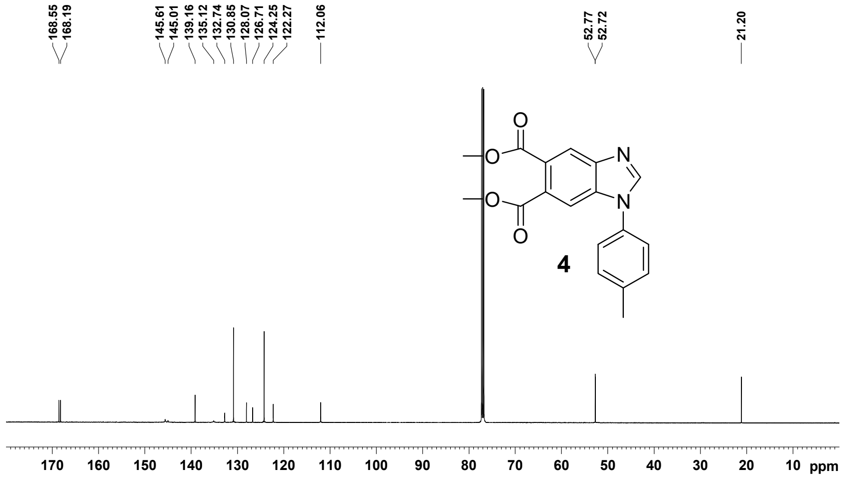

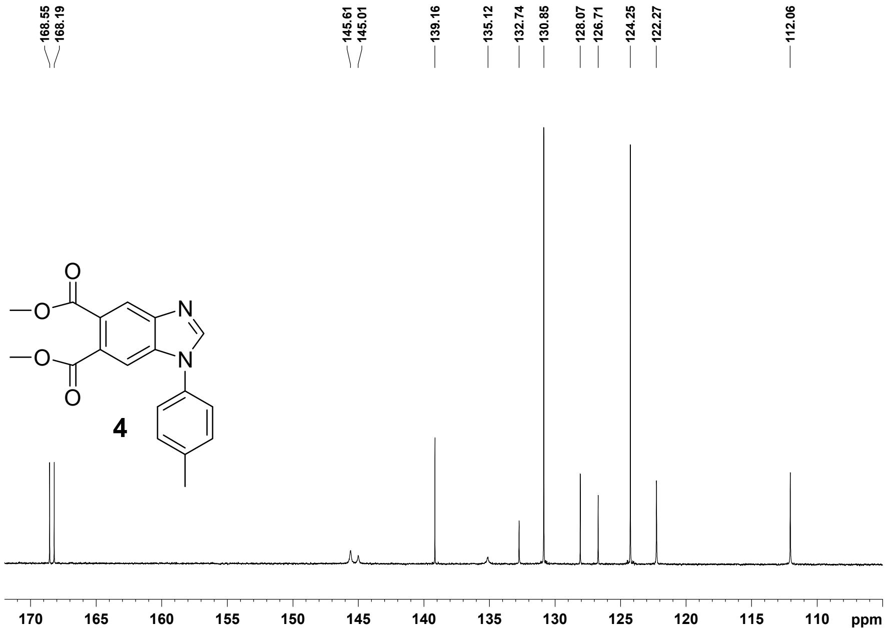

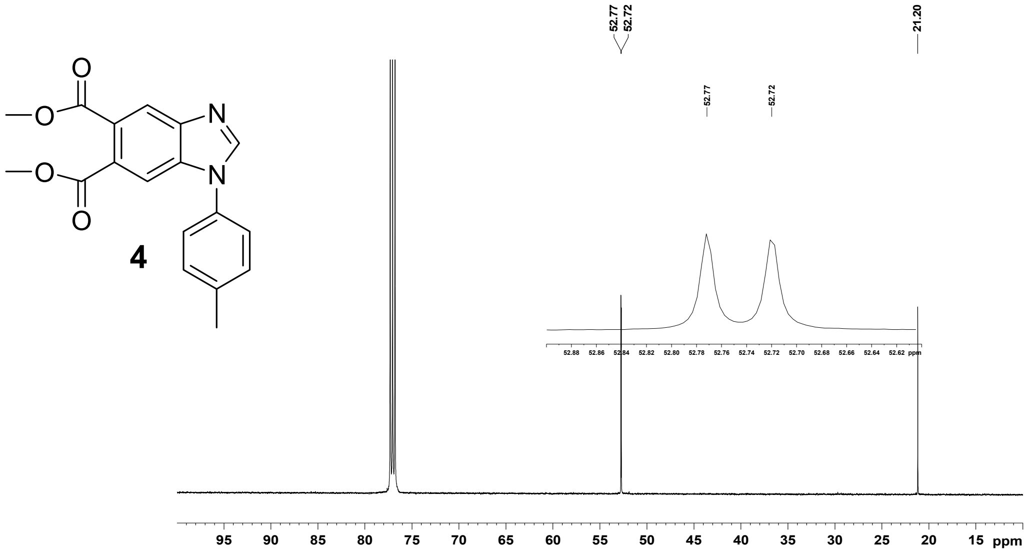

^{1}H-NMR (CDCl3) spectrum of **6**

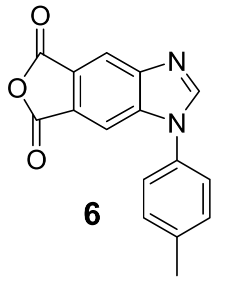

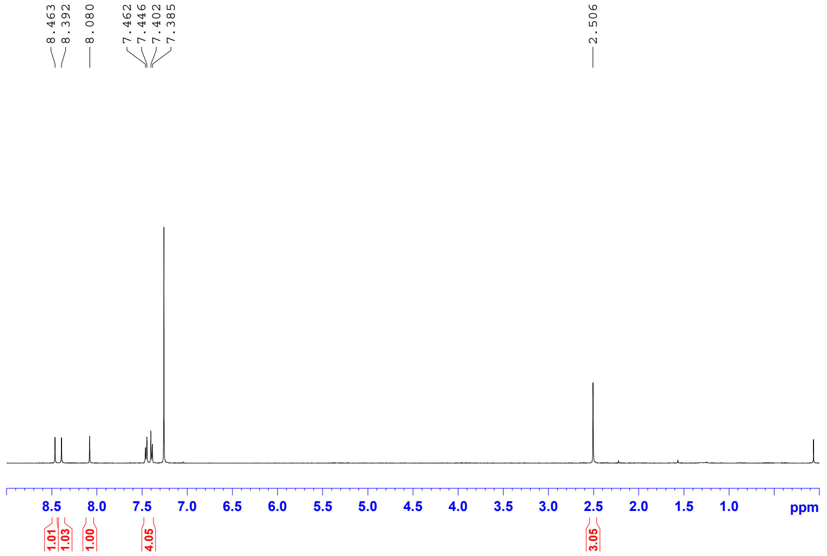
8.463
8.392
8.080
7.462
7.446
7.402
7.385
2.506
8.5 8.0 7.5 7.0 6.5 6.0 5.5 5.0 4.5 4.0 3.5 3.0 2.5 2.0 1.5 1.0 ppm
1.01
1.03
1.00
4.05
3.05

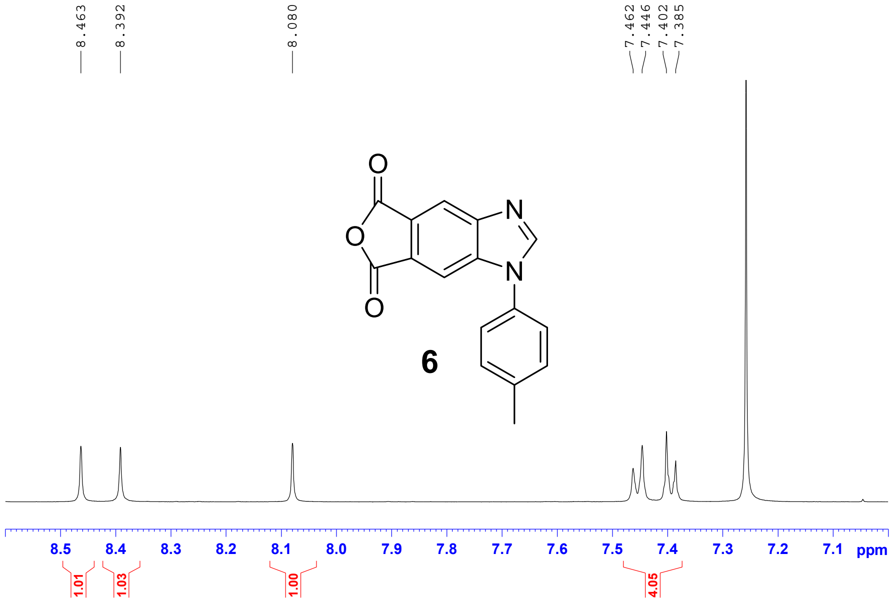
8.463
8.392
8.080
7.462
7.446
7.402
7.385
6
8.5 8.4 8.3 8.2 8.1 8.0 7.9 7.8 7.7 7.6 7.5 7.4 7.3 7.2 7.1 ppm
1.01
1.03
1.00
4.05

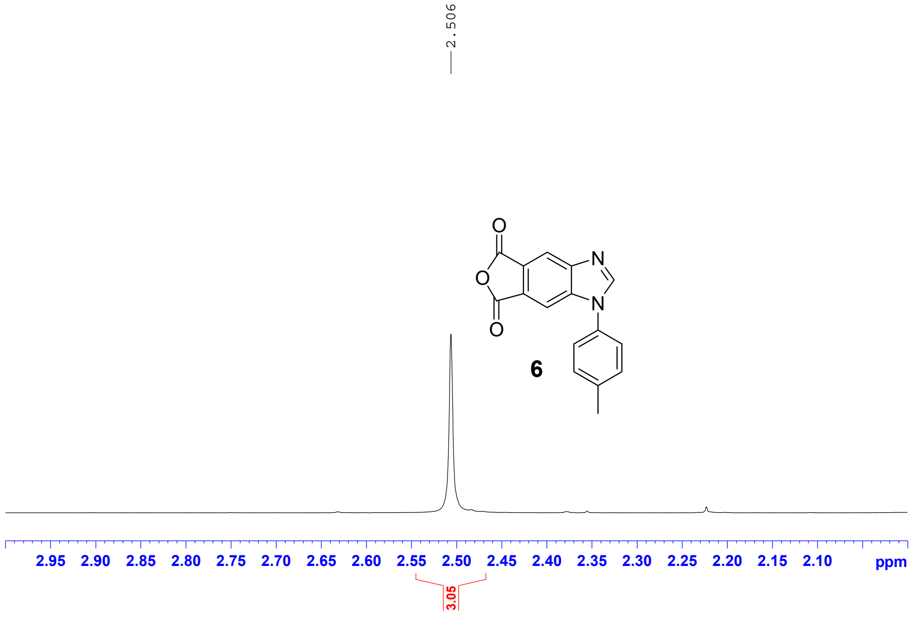
2.506
6
2.95 2.90 2.85 2.80 2.75 2.70 2.65 2.60 2.55 2.50 2.45 2.40 2.35 2.30 2.25 2.20 2.15 2.10 ppm
3.05

^{13}C-NMR (CDCl3) spectrum of **6**

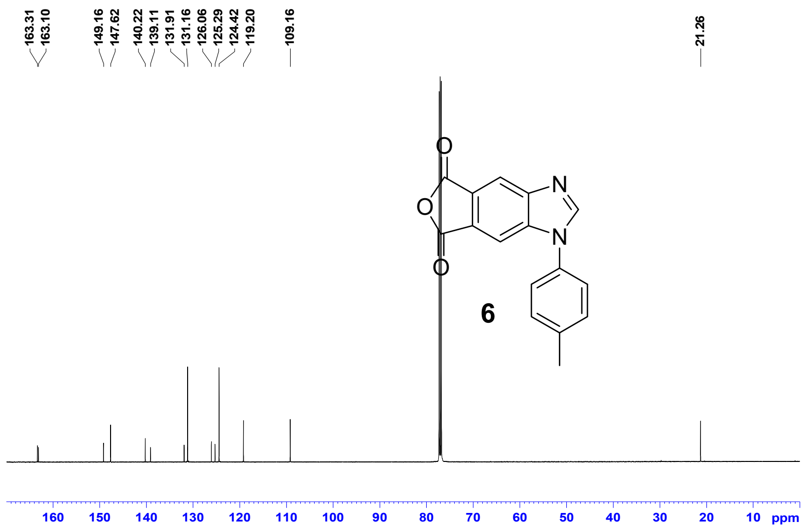

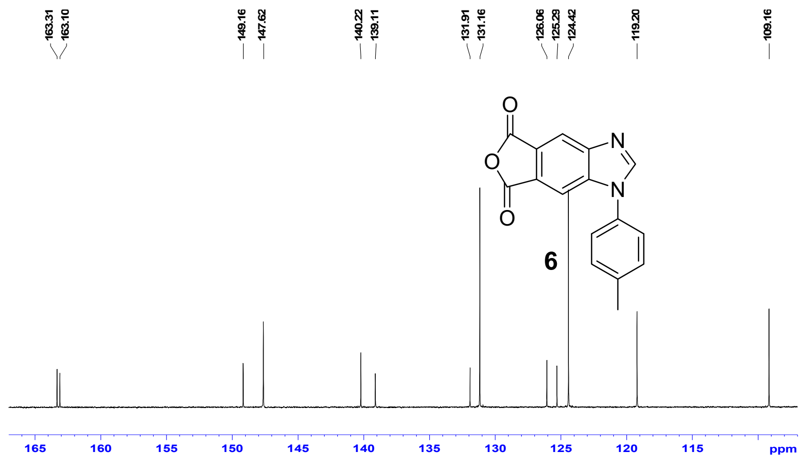

^{1}H-NMR (CDCl3) spectrum of **7**

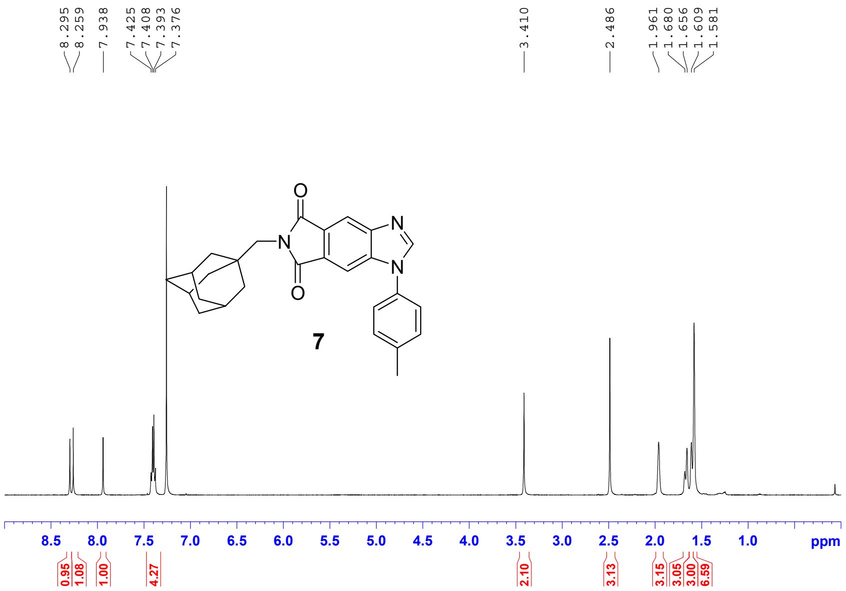

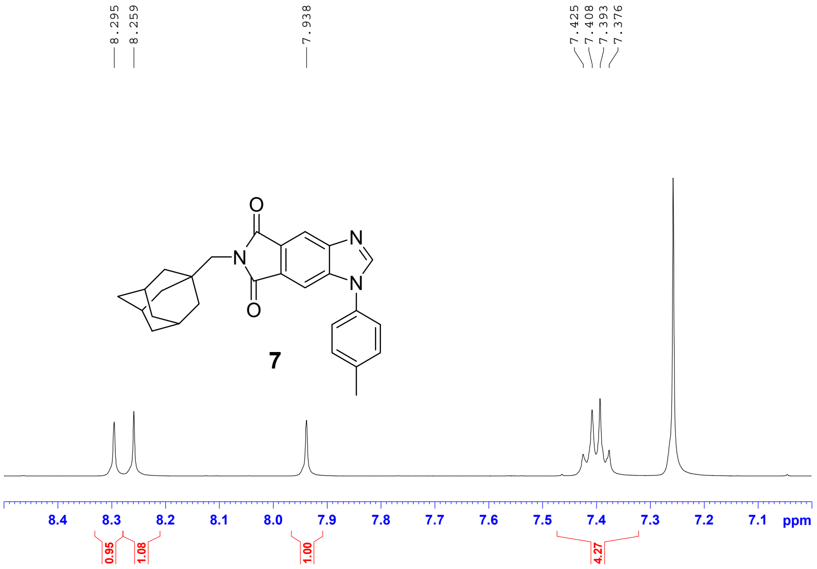

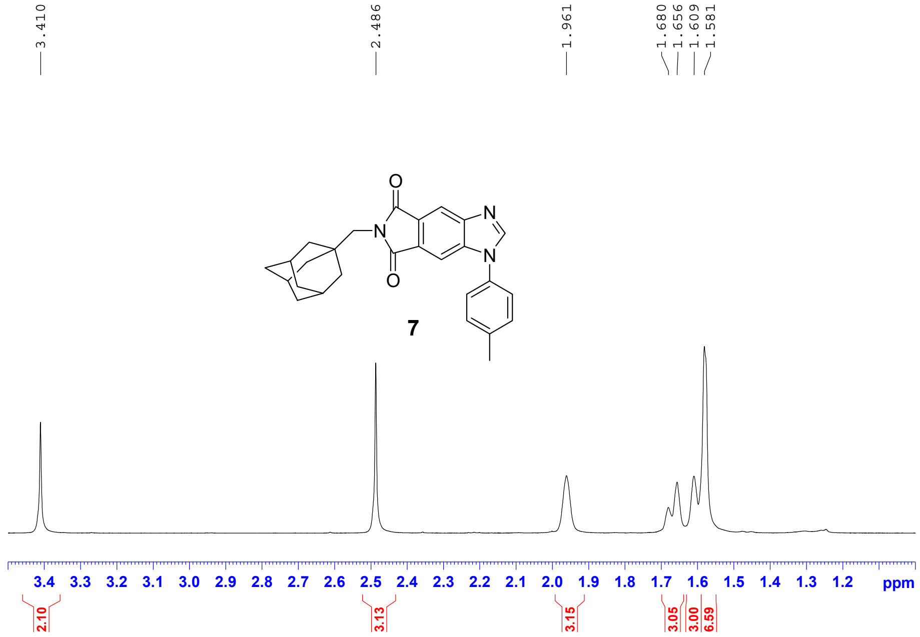

^{13}C-NMR (CDCl3) spectrum of **7**

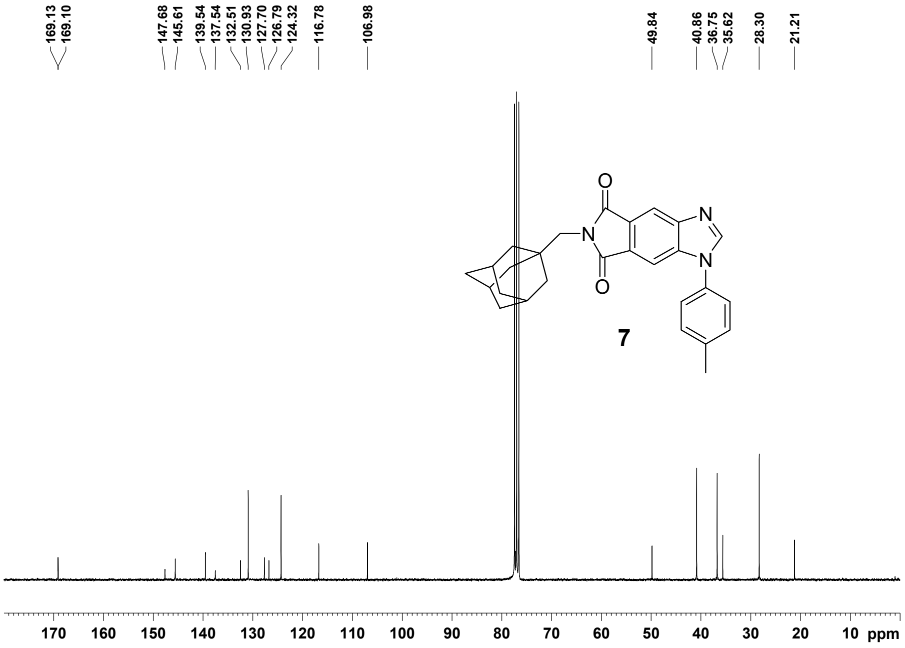

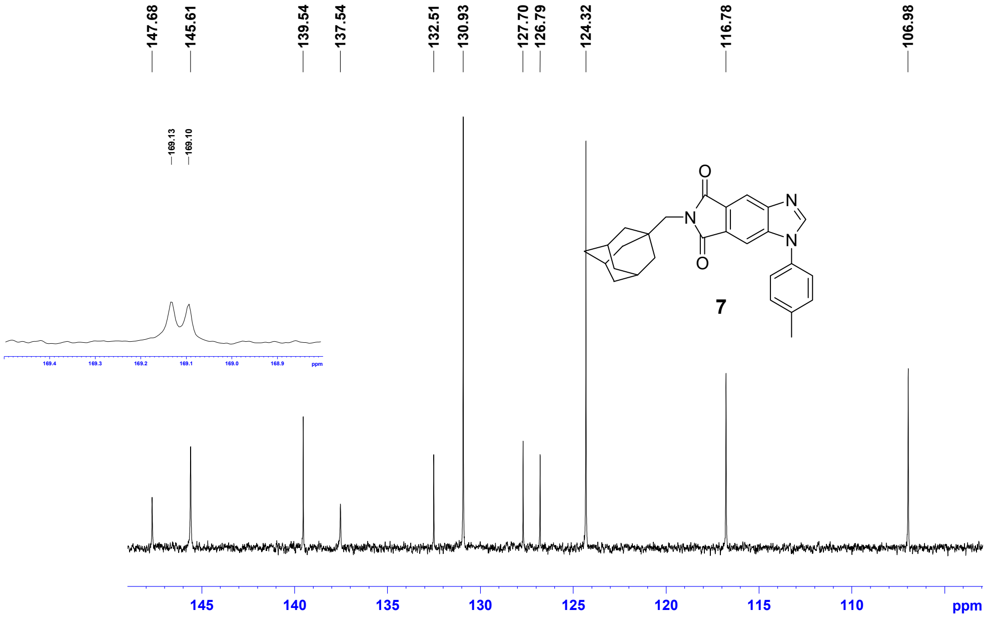
147.68
145.61
139.54
137.54
132.51
130.93
127.70
126.79
124.32
116.78
106.98
169.13
169.10
169.4
169.3
169.2
169.1
169.0
168.9
ppm
O
N
N
N
O
7
145
140
135
130
125
120
115
110
ppm

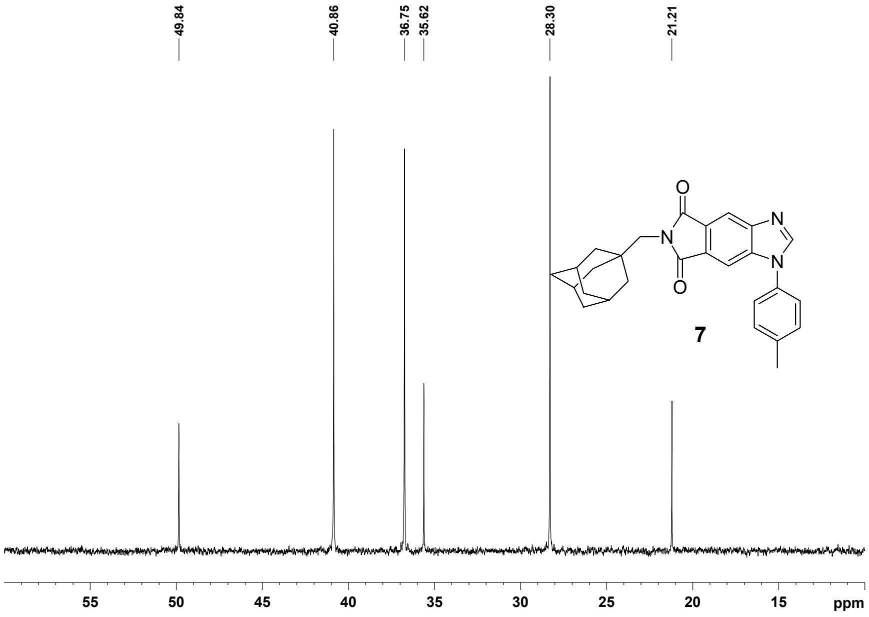
49.84
40.86
36.75
35.62
28.30
21.21
O
N
N
N
O
7
55
50
45
40
35
30
25
20
15
ppm

^{1}H-NMR (CD_3OD) spectrum of **8**

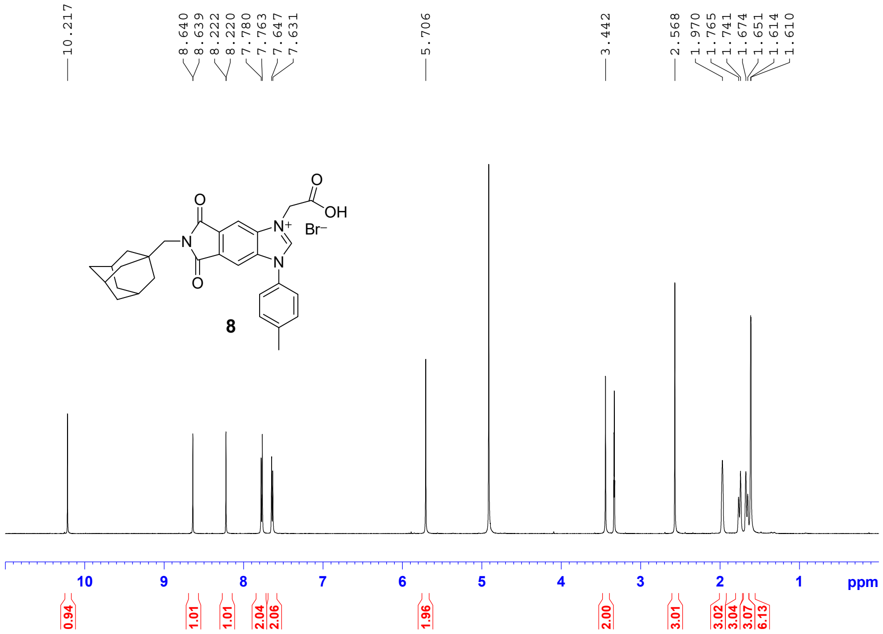

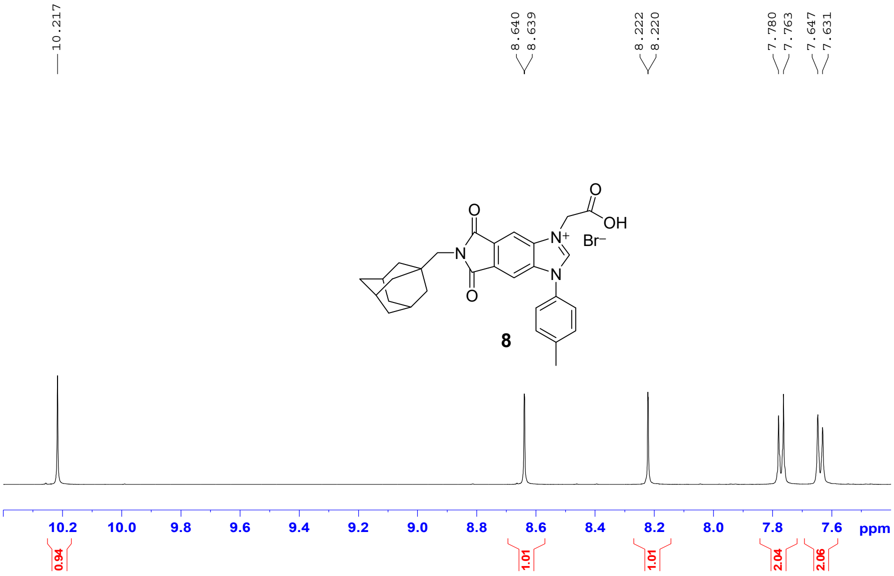

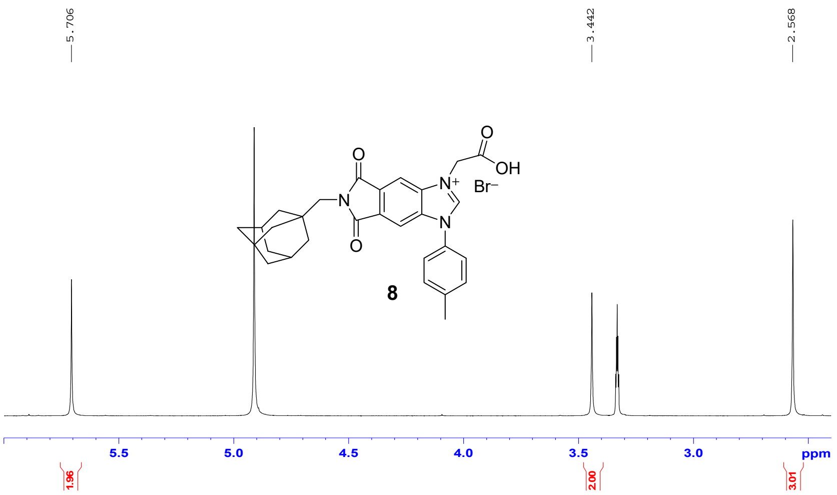

5.706
3.442
2.568
O
OH
O
N+
Br−
N
N
O
8
5.5
5.0
4.5
4.0
3.5
3.0
ppm
1.96
2.00
3.01

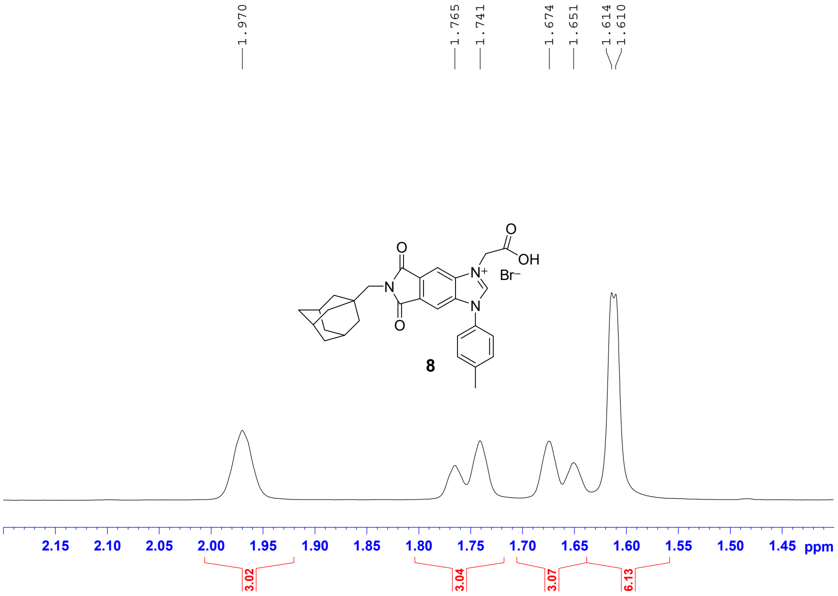

1.970
1.765
1.741
1.674
1.651
1.614
1.610
O
OH
O
N+
Br−
N
N
O
8
2.15
2.10
2.05
2.00
1.95
1.90
1.85
1.80
1.75
1.70
1.65
1.60
1.55
1.50
1.45
ppm
3.02
3.04
3.07
6.13

^{13}C-NMR (CD_3OD) spectrum of **8**

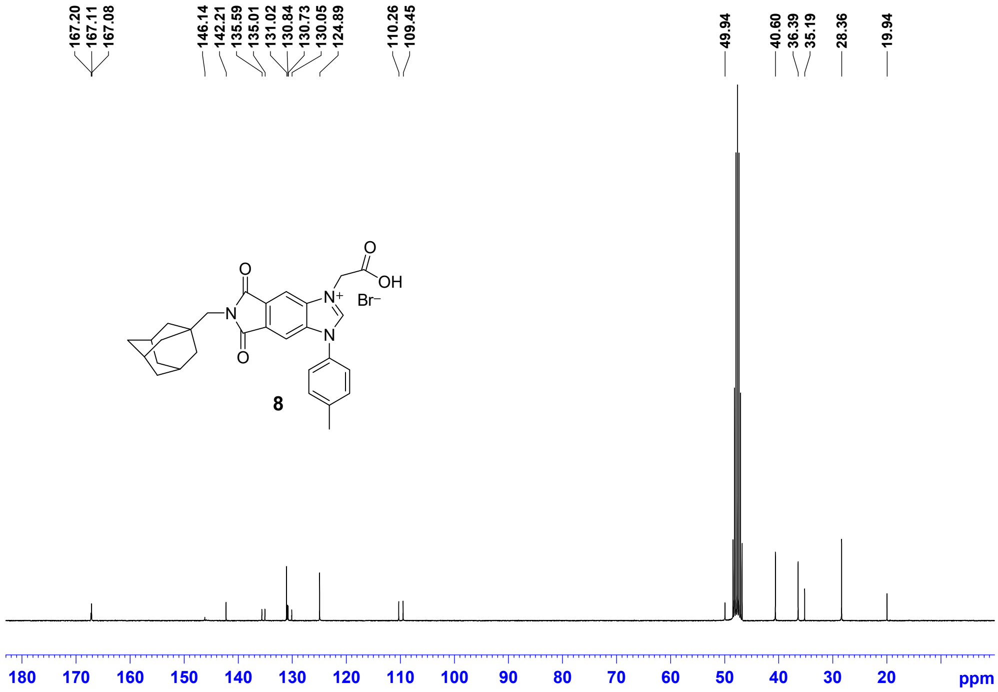

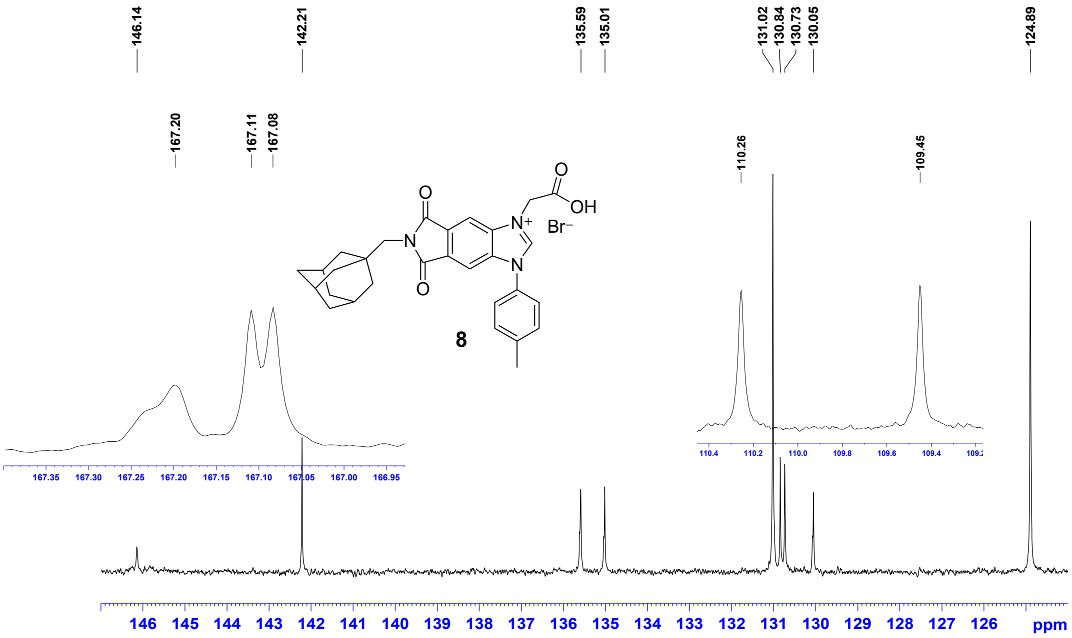

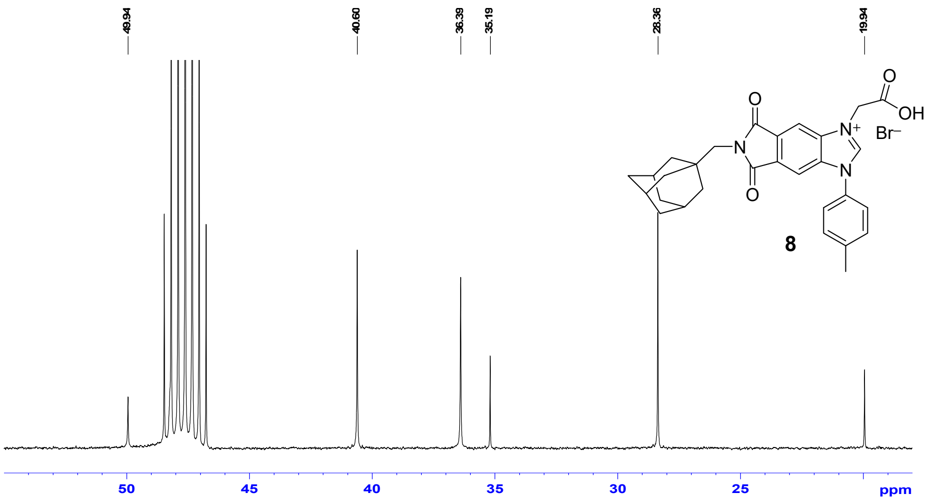

$C_{27}H_{27}N_3O_2 \cdot ½\ C_4H_{10}O$

Crystal Structure Report for D8_2824_HK_BTAdme (7)

A specimen of $C_{29}H_{32}N_3O_{2.50}$, approximate dimensions 0.065 mm x 0.319 mm x 0.322 mm, was used for the X-ray crystallographic analysis. The X-ray intensity data were measured.

The integration of the data using a monoclinic unit cell yielded a total of 58862 reflections to a maximum θ angle of 25.50° (0.83 Å resolution), of which 4472 were independent (average redundancy 13.162, completeness = 99.9%, R_{int} = 5.68%, R_{sig} = 2.10%) and 3671 (82.09%) were greater than $2\sigma(F^2)$. The final cell constants of a = 16.0391(9) Å, b = 12.5958(7) Å, c = 12.2622(6) Å, β = 104.282(2)°, volume = 2400.7(2) $Å^3$, are based upon the refinement of the XYZ-centroids of reflections above 20 σ(I). The calculated minimum and maximum transmission coefficients (based on crystal size) are 0.9305 and 1.0000.

The structure was solved and refined using the Bruker SHELXTL Software Package, using the space group P 1 21/c 1, with Z = 4 for the formula unit, $C_{29}H_{32}N_3O_{2.50}$. The final anisotropic full-matrix least-squares refinement on F^2 with 334 variables converged at R1 = 5.11%, for the observed data and wR2 = 13.18% for all data. The goodness-of-fit was 1.058. The largest peak in the final difference electron density synthesis was 0.403 $e^-/Å^3$ and the largest hole was -0.264 $e^-/Å^3$ with an RMS deviation of 0.048 $e^-/Å^3$. On the basis of the final model, the calculated density was 1.280 g/cm^3 and F(000), 988 e^-.

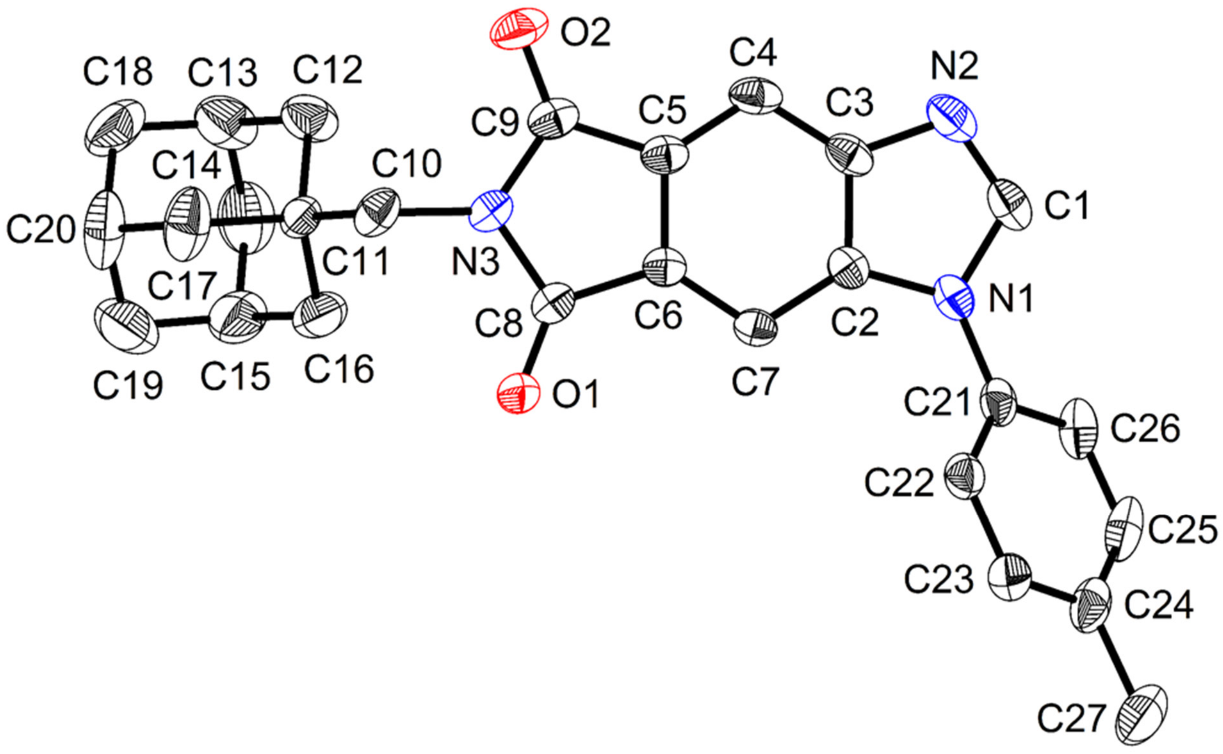
C18
C13
C12
O2
C4
N2
C9
C5
C3
C14
C10
C20
C1
C11
N3
C17
N1
C8
C6
C2
C19
C15
C16
O1
C7
C21
C26
C22
C25
C23
C24
C27

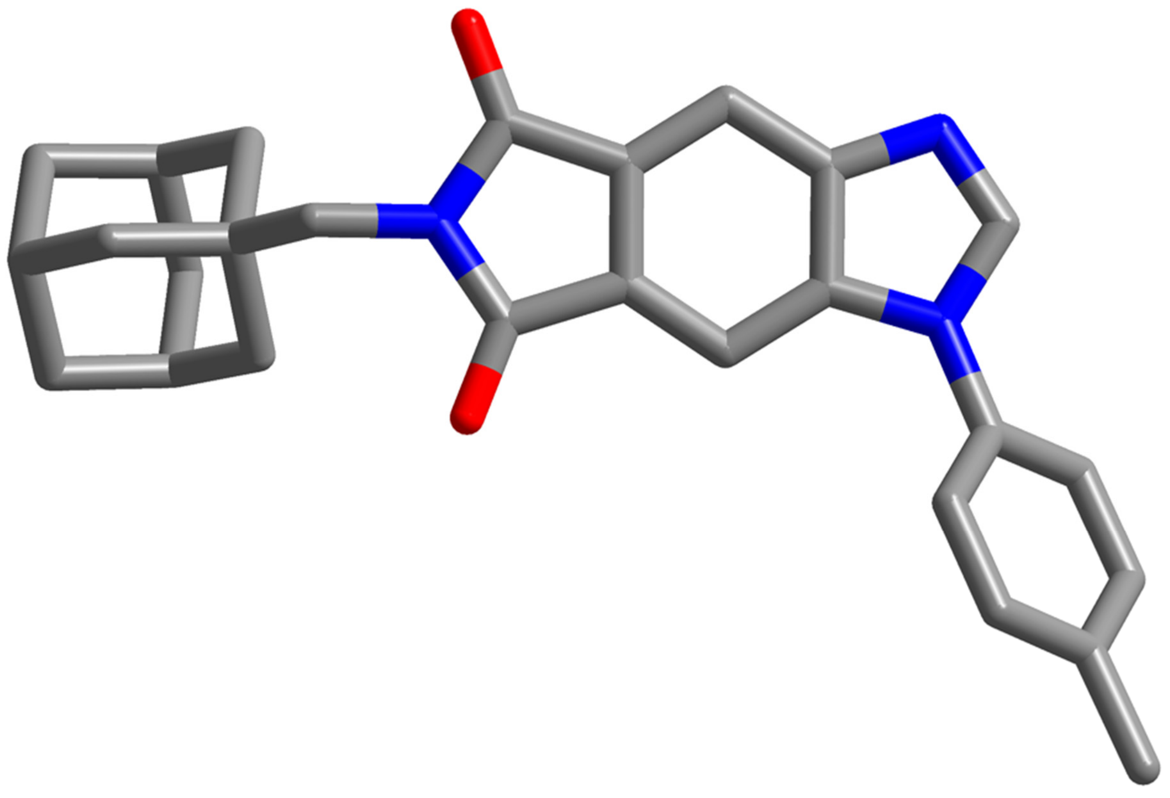

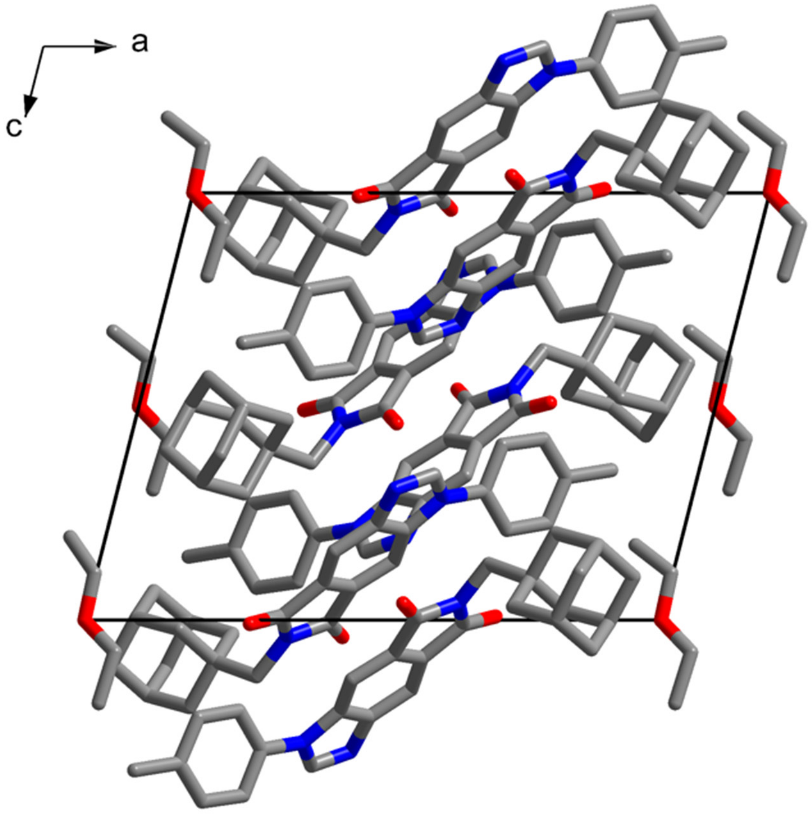

a
c

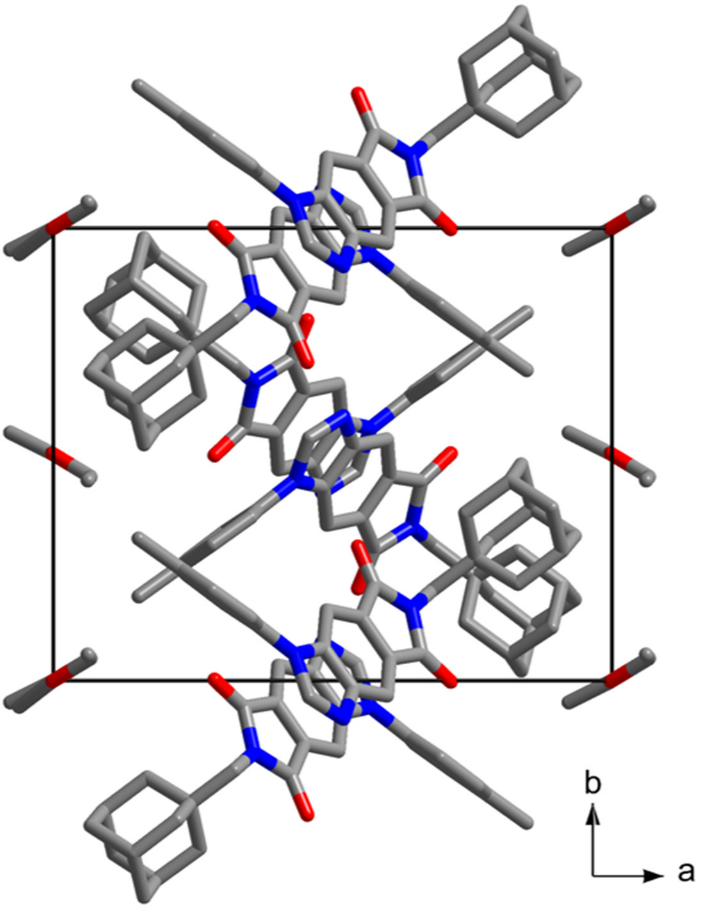
b
a

Table S1. Sample and crystal data for D8_2824_HK_BTolAd.

**Identification code**	D8_2824_HK_BTolAd	
**Chemical formula**	$C_{29}H_{32}N_3O_{2.50}$	
**Formula weight**	462.57 g/mol	
**Temperature**	140(2) K	
**Wavelength**	0.71073 Å	
**Crystal size**	0.065 x 0.319 x 0.322 mm	
**Crystal system**	monoclinic	
**Space group**	P 1 21/c 1	
**Unit cell dimensions**	a = 16.0391(9) Å	α = 90°
	b = 12.5958(7) Å	β = 104.282(2)°
	c = 12.2622(6) Å	γ = 90°
**Volume**	2400.7(2) $Å^3$	
**Z**	4	
**Density (calculated)**	1.280 g/cm^3	
**Absorption coefficient**	0.082 mm^{-1}	
**F(000)**	988	

Table S2. Data collection and structure refinement for D8_2824_HK_BTolAd.

**Theta range for data collection**	2.36 to 25.50°
**Index ranges**	-19<=h<=19, -15<=k<=15, -14<=l<=14
**Reflections collected**	58862
**Independent reflections**	4472 [R(int) = 0.0568]
**Max. and min. transmission**	1.0000 and 0.9305
**Structure solution technique**	direct methods
**Structure solution program**	SHELXT-2014 (Sheldrick 2014)
**Refinement method**	Full-matrix least-squares on F^2
**Refinement program**	SHELXL-2014 (Sheldrick 2014)

**Function minimized**	Σ w(F_o^2 - F_c^2)^2	
**Data / restraints / parameters**	4472 / 0 / 334	
**Goodness-of-fit on F^2**	1.058	
**Final R indices**	3671 data; I>2σ(I)	R1 = 0.0511, wR2 = 0.1230
	all data	R1 = 0.0637, wR2 = 0.1318
**Weighting scheme**	w=1/[σ^2(F_o^2)+(0.0542P)^2+1.5599P]<br>where P=(F_o^2+2F_c^2)/3	
**Largest diff. peak and hole**	0.403 and -0.264 eÅ^{-3}	
**R.M.S. deviation from mean**	0.048 eÅ^{-3}	

Table S3. Atomic coordinates and equivalent isotropic atomic displacement parameters (Å^2) for D8_2824_HK_BTolAd.

U(eq) is defined as one third of the trace of the orthogonalized U_{ij} tensor.

	**x/a**	**y/b**	**z/c**	**U(eq)**
O1	0.54538(8)	0.20374(11)	0.46052(11)	0.0360(3)
O2	0.71146(9)	0.50230(11)	0.49131(13)	0.0440(4)
N1	0.43196(10)	0.44201(12)	0.78604(12)	0.0295(3)
N2	0.51934(11)	0.58422(13)	0.80781(14)	0.0405(4)
N3	0.64102(9)	0.34088(12)	0.45957(12)	0.0299(3)
C1	0.45760(13)	0.53589(15)	0.84042(17)	0.0374(5)
C2	0.48333(11)	0.42792(14)	0.71130(14)	0.0261(4)
C3	0.53674(12)	0.51878(14)	0.72533(15)	0.0311(4)
C4	0.59725(11)	0.53296(14)	0.66150(16)	0.0332(4)
C5	0.60216(11)	0.45196(14)	0.58833(15)	0.0285(4)
C6	0.55065(11)	0.36046(13)	0.57830(14)	0.0260(4)
C7	0.48969(11)	0.34474(14)	0.63858(14)	0.0265(4)
C8	0.57473(11)	0.28960(15)	0.49462(14)	0.0284(4)
C9	0.65904(11)	0.44056(15)	0.51035(15)	0.0315(4)
C10	0.67671(12)	0.30176(17)	0.36853(15)	0.0356(4)

	x/a	y/b	z/c	U(eq)
C11	0.75499(11)	0.22918(15)	0.40383(15)	0.0307(4)
C12	0.83170(13)	0.28616(17)	0.4790(2)	0.0468(5)
C13	0.91080(14)	0.2130(2)	0.5057(2)	0.0571(7)
C14	0.89225(16)	0.1178(2)	0.5681(2)	0.0629(7)
C15	0.81784(16)	0.05821(19)	0.4977(2)	0.0597(7)
C16	0.73750(14)	0.12994(17)	0.4684(2)	0.0495(6)
C17	0.77902(16)	0.1923(2)	0.29651(19)	0.0603(7)
C18	0.93354(15)	0.1795(2)	0.3984(3)	0.0644(7)
C19	0.83787(18)	0.0223(2)	0.3883(3)	0.0709(8)
C20	0.85718(17)	0.1185(3)	0.3237(2)	0.0668(8)
C21	0.36381(12)	0.37586(14)	0.80273(15)	0.0299(4)
C22	0.30750(12)	0.32982(15)	0.71127(15)	0.0327(4)
C23	0.24042(13)	0.26727(16)	0.72788(17)	0.0383(5)
C24	0.22711(13)	0.25190(16)	0.83424(18)	0.0404(5)
C25	0.28394(13)	0.30027(18)	0.92416(17)	0.0430(5)
C26	0.35261(13)	0.36070(17)	0.91024(16)	0.0383(5)
C27	0.15254(15)	0.1861(2)	0.8512(2)	0.0574(7)
O30	0.0	0.5	0.5	0.0600(6)
C29	0.0033(3)	0.4975(4)	0.6110(4)	0.0537(12)
C28	0.0612(11)	0.4424(12)	0.6943(13)	0.099(5)
C29B	0.0665(3)	0.4506(3)	0.5746(4)	0.0465(11)
C28B	0.0772(9)	0.4520(14)	0.6755(11)	0.108(6)

Table S4. Bond lengths (Å) for D8_2824_HK_BTolAd.

O1-C8	1.212(2)	O2-C9	1.210(2)
N1-C1	1.370(2)	N1-C2	1.387(2)
N1-C21	1.429(2)	N2-C1	1.306(3)

N2-C3	1.386(2)	N3-C8	1.399(2)
N3-C9	1.400(2)	N3-C10	1.460(2)
C1-H1	0.95	C2-C7	1.396(2)
C2-C3	1.414(3)	C3-C4	1.400(3)
C4-C5	1.374(3)	C4-H4	0.95
C5-C6	1.406(2)	C5-C9	1.483(3)
C6-C7	1.378(2)	C6-C8	1.481(2)
C7-H7	0.95	C10-C11	1.527(3)
C10-H10A	0.99	C10-H10B	0.99
C11-C12	1.523(3)	C11-C17	1.532(3)
C11-C16	1.542(3)	C12-C13	1.537(3)
C12-H12A	0.99	C12-H12B	0.99
C13-C14	1.491(4)	C13-C18	1.510(4)
C13-H13	1.0	C14-C15	1.491(4)
C14-H14A	0.99	C14-H14B	0.99
C15-C19	1.523(4)	C15-C16	1.542(3)
C15-H15	1.0	C16-H16A	0.99
C16-H16B	0.99	C17-C20	1.530(4)
C17-H17A	0.99	C17-H17B	0.99
C18-C20	1.541(4)	C18-H18A	0.99
C18-H18B	0.99	C19-C20	1.521(4)
C19-H19A	0.99	C19-H19B	0.99
C20-H20	1.0	C21-C22	1.382(3)
C21-C26	1.387(3)	C22-C23	1.388(3)
C22-H22	0.95	C23-C24	1.386(3)
C23-H23	0.95	C24-C25	1.386(3)
C24-C27	1.511(3)	C25-C26	1.384(3)
C25-H25	0.95	C26-H26	0.95
C27-H27A	0.98	C27-H27B	0.98

C27-H27C	0.98	O30-C29	1.349(5)
O30-C29	1.349(5)	O30-C29B	1.371(5)
O30-C29B	1.371(5)	C29-C28	1.387(16)
C29-H29A	0.99	C29-H29B	0.99
C28-H28A	0.98	C28-H28B	0.98
C28-H28C	0.98	C29B-C28B	1.206(14)
C29B-H29C	0.99	C29B-H29D	0.99
C28B-H28D	0.98	C28B-H28E	0.98
C28B-H28F	0.98		

Table S5. Bond angles (°) for D8_2824_HK_BTolAd.

C1-N1-C2	106.03(16)	C1-N1-C21	125.60(16)
C2-N1-C21	128.34(15)	C1-N2-C3	104.52(16)
C8-N3-C9	111.51(15)	C8-N3-C10	123.37(15)
C9-N3-C10	124.47(15)	N2-C1-N1	114.37(17)
N2-C1-H1	122.8	N1-C1-H1	122.8
N1-C2-C7	131.86(16)	N1-C2-C3	104.89(15)
C7-C2-C3	123.20(16)	N2-C3-C4	128.63(17)
N2-C3-C2	110.19(17)	C4-C3-C2	121.18(17)
C5-C4-C3	115.44(16)	C5-C4-H4	122.3
C3-C4-H4	122.3	C4-C5-C6	122.62(17)
C4-C5-C9	129.55(17)	C6-C5-C9	107.82(15)
C7-C6-C5	123.39(16)	C7-C6-C8	128.75(16)
C5-C6-C8	107.86(15)	C6-C7-C2	114.10(16)
C6-C7-H7	122.9	C2-C7-H7	122.9
O1-C8-N3	124.46(16)	O1-C8-C6	129.14(16)
N3-C8-C6	106.40(15)	O2-C9-N3	124.61(18)
O2-C9-C5	129.05(18)	N3-C9-C5	106.34(14)
N3-C10-C11	115.81(15)	N3-C10-H10A	108.3

C11-C10-H10A	108.3	N3-C10-H10B	108.3
C11-C10-H10B	108.3	H10A-C10-H10B	107.4
C12-C11-C10	111.99(16)	C12-C11-C17	109.03(17)
C10-C11-C17	107.55(16)	C12-C11-C16	107.06(17)
C10-C11-C16	112.96(15)	C17-C11-C16	108.16(19)
C11-C12-C13	110.34(17)	C11-C12-H12A	109.6
C13-C12-H12A	109.6	C11-C12-H12B	109.6
C13-C12-H12B	109.6	H12A-C12-H12B	108.1
C14-C13-C18	110.1(2)	C14-C13-C12	109.4(2)
C18-C13-C12	110.3(2)	C14-C13-H13	109.0
C18-C13-H13	109.0	C12-C13-H13	109.0
C13-C14-C15	109.9(2)	C13-C14-H14A	109.7
C15-C14-H14A	109.7	C13-C14-H14B	109.7
C15-C14-H14B	109.7	H14A-C14-H14B	108.2
C14-C15-C19	110.3(2)	C14-C15-C16	110.1(2)
C19-C15-C16	108.3(2)	C14-C15-H15	109.4
C19-C15-H15	109.4	C16-C15-H15	109.4
C15-C16-C11	110.34(17)	C15-C16-H16A	109.6
C11-C16-H16A	109.6	C15-C16-H16B	109.6
C11-C16-H16B	109.6	H16A-C16-H16B	108.1
C20-C17-C11	111.28(19)	C20-C17-H17A	109.4
C11-C17-H17A	109.4	C20-C17-H17B	109.4
C11-C17-H17B	109.4	H17A-C17-H17B	108.0
C13-C18-C20	109.24(18)	C13-C18-H18A	109.8
C20-C18-H18A	109.8	C13-C18-H18B	109.8
C20-C18-H18B	109.8	H18A-C18-H18B	108.3
C20-C19-C15	109.7(2)	C20-C19-H19A	109.7
C15-C19-H19A	109.7	C20-C19-H19B	109.7
C15-C19-H19B	109.7	H19A-C19-H19B	108.2

C19-C20-C17	109.7(2)	C19-C20-C18	108.8(2)
C17-C20-C18	108.3(2)	C19-C20-H20	110.0
C17-C20-H20	110.0	C18-C20-H20	110.0
C22-C21-C26	120.11(18)	C22-C21-N1	119.89(16)
C26-C21-N1	119.96(17)	C21-C22-C23	119.60(17)
C21-C22-H22	120.2	C23-C22-H22	120.2
C24-C23-C22	121.5(2)	C24-C23-H23	119.3
C22-C23-H23	119.3	C23-C24-C25	117.61(19)
C23-C24-C27	121.1(2)	C25-C24-C27	121.3(2)
C26-C25-C24	122.06(18)	C26-C25-H25	119.0
C24-C25-H25	119.0	C25-C26-C21	119.12(19)
C25-C26-H26	120.4	C21-C26-H26	120.4
C24-C27-H27A	109.5	C24-C27-H27B	109.5
H27A-C27-H27B	109.5	C24-C27-H27C	109.5
H27A-C27-H27C	109.5	H27B-C27-H27C	109.5
C29-O30-C29	180.0(4)	C29B-O30-C29B	180.0
O30-C29-C28	127.4(7)	O30-C29-H29A	105.5
C28-C29-H29A	105.5	O30-C29-H29B	105.5
C28-C29-H29B	105.5	H29A-C29-H29B	106.0
C29-C28-H28A	109.5	C29-C28-H28B	109.5
H28A-C28-H28B	109.5	C29-C28-H28C	109.5
H28A-C28-H28C	109.5	H28B-C28-H28C	109.5
C28B-C29B-O30	124.7(8)	C28B-C29B-H29C	106.2
O30-C29B-H29C	106.2	C28B-C29B-H29D	106.2
O30-C29B-H29D	106.2	H29C-C29B-H29D	106.4
C29B-C28B-H28D	109.5	C29B-C28B-H28E	109.5
H28D-C28B-H28E	109.5	C29B-C28B-H28F	109.5
H28D-C28B-H28F	109.5	H28E-C28B-H28F	109.5

Table S6. Anisotropic atomic displacement parameters (Å²) for D8_2824_HK_BTolAd.

The anisotropic atomic displacement factor exponent takes the form: $-2\pi^2[h^2 a^{*2} U_{11} + ... + 2 h k a^* b^* U_{12}]$

	U_{11}	U_{22}	U_{33}	U_{23}	U_{13}	U_{12}
O1	0.0340(7)	0.0376(8)	0.0396(7)	-0.0112(6)	0.0156(6)	-0.0112(6)
O2	0.0383(8)	0.0346(8)	0.0620(10)	0.0132(7)	0.0180(7)	-0.0071(6)
N1	0.0344(8)	0.0253(8)	0.0282(8)	-0.0015(6)	0.0066(6)	0.0052(6)
N2	0.0418(9)	0.0283(9)	0.0480(10)	-0.0102(7)	0.0046(8)	0.0028(7)
N3	0.0263(7)	0.0336(8)	0.0310(8)	0.0040(7)	0.0095(6)	-0.0030(6)
C1	0.0409(11)	0.0311(10)	0.0381(10)	-0.0088(8)	0.0058(8)	0.0078(9)
C2	0.0290(9)	0.0226(9)	0.0254(8)	0.0033(7)	0.0038(7)	0.0051(7)
C3	0.0332(9)	0.0208(9)	0.0352(10)	-0.0003(7)	0.0007(8)	0.0045(7)
C4	0.0293(9)	0.0212(9)	0.0450(11)	0.0049(8)	0.0014(8)	-0.0017(7)
C5	0.0272(9)	0.0232(9)	0.0329(9)	0.0070(7)	0.0032(7)	0.0003(7)
C6	0.0264(9)	0.0242(9)	0.0260(8)	0.0035(7)	0.0036(7)	0.0005(7)
C7	0.0295(9)	0.0210(8)	0.0281(9)	0.0019(7)	0.0057(7)	-0.0012(7)
C8	0.0245(8)	0.0327(10)	0.0280(9)	0.0031(8)	0.0065(7)	-0.0022(7)
C9	0.0258(9)	0.0295(10)	0.0369(10)	0.0113(8)	0.0037(7)	0.0002(8)
C10	0.0305(9)	0.0503(12)	0.0272(9)	0.0033(8)	0.0096(7)	-0.0045(9)
C11	0.0270(9)	0.0379(11)	0.0292(9)	-0.0034(8)	0.0104(7)	-0.0060(8)
C12	0.0335(11)	0.0387(12)	0.0622(14)	-0.0100(10)	0.0006(9)	-0.0020(9)
C13	0.0328(11)	0.0497(14)	0.0792(17)	-0.0156(13)	-0.0048(11)	0.0013(10)
C14	0.0549(15)	0.0677(17)	0.0643(16)	0.0046(13)	0.0110(12)	0.0237(13)
C15	0.0594(15)	0.0357(12)	0.093(2)	0.0141(13)	0.0361(14)	0.0048(11)
C16	0.0422(12)	0.0398(12)	0.0722(16)	0.0048(11)	0.0251(11)	-0.0049(10)
C17	0.0524(14)	0.094(2)	0.0373(12)	-0.0062(13)	0.0170(10)	0.0113(14)
C18	0.0365(12)	0.0595(16)	0.106(2)	0.0144(15)	0.0341(13)	0.0034(11)
C19	0.0548(15)	0.0474(15)	0.107(2)	-0.0268(15)	0.0132(15)	-0.0022(12)
C20	0.0550(15)	0.101(2)	0.0510(14)	-0.0143(14)	0.0247(12)	0.0208(15)

	U_{11}	U_{22}	U_{33}	U_{23}	U_{13}	U_{12}
C21	0.0346(10)	0.0256(9)	0.0323(9)	0.0028(8)	0.0135(8)	0.0105(8)
C22	0.0410(10)	0.0306(10)	0.0299(9)	-0.0005(8)	0.0150(8)	0.0016(8)
C23	0.0411(11)	0.0338(11)	0.0441(11)	-0.0022(9)	0.0183(9)	0.0007(9)
C24	0.0401(11)	0.0365(11)	0.0521(12)	0.0118(9)	0.0258(10)	0.0148(9)
C25	0.0447(12)	0.0540(13)	0.0382(11)	0.0158(10)	0.0251(9)	0.0228(10)
C26	0.0393(11)	0.0473(12)	0.0295(10)	0.0012(9)	0.0109(8)	0.0170(9)
C27	0.0520(14)	0.0553(15)	0.0774(17)	0.0173(13)	0.0394(13)	0.0085(11)
O30	0.0479(13)	0.0718(17)	0.0630(16)	0.0057(13)	0.0190(11)	0.0097(12)
C29	0.058(3)	0.045(3)	0.056(3)	-0.007(2)	0.011(2)	0.004(2)
C28	0.140(9)	0.084(8)	0.085(8)	-0.018(7)	0.053(7)	-0.039(6)
C29B	0.046(2)	0.032(2)	0.067(3)	0.005(2)	0.025(2)	0.0021(19)
C28B	0.103(8)	0.124(9)	0.069(6)	0.061(6)	-0.031(6)	-0.034(7)

Table S7. Hydrogen atomic coordinates and isotropic atomic displacement parameters (Å^2) for D8_2824_HK_BTolAd.

	x/a	y/b	z/c	U(eq)
H1	0.4326	0.5635	0.8971	0.045
H4	0.6325	0.5944	0.6683	0.04
H7	0.4550	0.2827	0.6313	0.032
H10A	0.6929	0.3637	0.3284	0.043
H10B	0.6310	0.2628	0.3144	0.043
H12A	0.8175	0.3078	0.5499	0.056
H12B	0.8448	0.3510	0.4407	0.056
H13	0.9606	0.2523	0.5540	0.069
H14A	0.8789	0.1403	0.6393	0.076
H14B	0.9435	0.0712	0.5869	0.076
H15	0.8057	-0.0053	0.5403	0.072
H16A	0.7220	0.1519	0.5384	0.059

	x/a	y/b	z/c	U(eq)
H16B	0.6885	0.0898	0.4216	0.059
H17A	0.7295	0.1546	0.2476	0.072
H17B	0.7920	0.2550	0.2548	0.072
H18A	0.9852	0.1336	0.4163	0.077
H18B	0.9466	0.2429	0.3578	0.077
H19A	0.8881	-0.0260	0.4051	0.085
H19B	0.7881	-0.0169	0.3420	0.085
H20	0.8717	0.0953	0.2526	0.08
H22	0.3146	0.3409	0.6375	0.039
H23	0.2028	0.2343	0.6650	0.046
H25	0.2755	0.2916	0.9976	0.052
H26	0.3916	0.3914	0.9735	0.046
H27A	0.1740	0.1178	0.8850	0.086
H27B	0.1118	0.1739	0.7784	0.086
H27C	0.1235	0.2239	0.9012	0.086
H29A	-0.0544	0.4734	0.6161	0.064
H29B	0.0080	0.5726	0.6356	0.064
H28A	0.0774	0.3760	0.6631	0.148
H28B	0.0346	0.4262	0.7562	0.148
H28C	0.1127	0.4858	0.7224	0.148
H29C	0.0634	0.3748	0.5526	0.056
H29D	0.1200	0.4789	0.5593	0.056
H28D	0.0561	0.5194	0.6983	0.162
H28E	0.1387	0.4446	0.7114	0.162
H28F	0.0456	0.3931	0.6987	0.162

$C_{29}H_{30}BrN_3O_4 \cdot 0.5C_5H_{12}$

Crystal Structure Report for D8_2953_HK_BTolBr (8)

A specimen of $C_{31.50}H_{36}BrN_3O_4$, approximate dimensions 0.051 mm x 0.202 mm x 0.265 mm, was used for the X-ray crystallographic analysis. The X-ray intensity data were measured.

The integration of the data using an orthorhombic unit cell yielded a total of 38548 reflections to a maximum θ angle of 25.50° (0.83 Å resolution), of which 5780 were independent (average redundancy 6.669, completeness = 99.6%, R_{int} = 8.39%, R_{sig} = 5.16%) and 4770 (82.53%) were greater than $2\sigma(F^2)$. The final cell constants of a = 12.5299(12) Å, b = 49.749(5) Å, c = 10.0582(9) Å, volume = 6269.8(10) $Å^3$, are based upon the refinement of the XYZ-centroids of reflections above 20 σ(I). The calculated minimum and maximum transmission coefficients (based on crystal size) are 0.7241 and 1.0000.

The structure was solved and refined using the Bruker SHELXTL Software Package, using the space group I b a 2, with Z = 8 for the formula unit, $C_{31.50}H_{36}BrN_3O_4$. The final anisotropic full-matrix least-squares refinement on F^2 with 357 variables converged at R1 = 6.48%, for the observed data and wR2 = 17.40% for all data. The goodness-of-fit was 1.104. The largest peak in the final difference electron density synthesis was 0.825 $e^-/Å^3$ and the largest hole was -0.594 $e^-/Å^3$ with an RMS deviation of 0.103 $e^-/Å^3$. On the basis of the final model, the calculated density was 1.272 g/cm^3 and F(000), 2504 e^-.

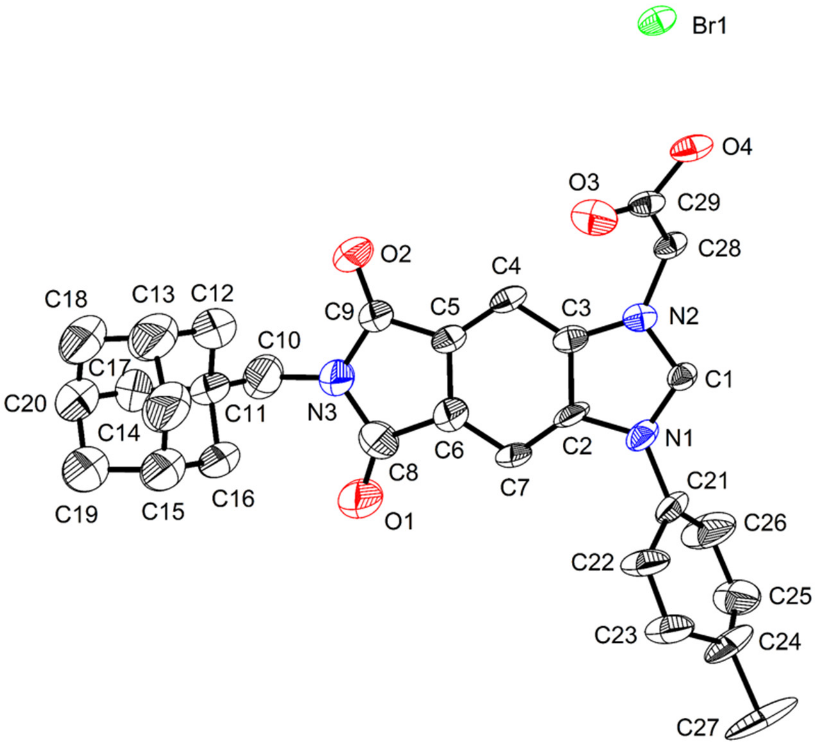

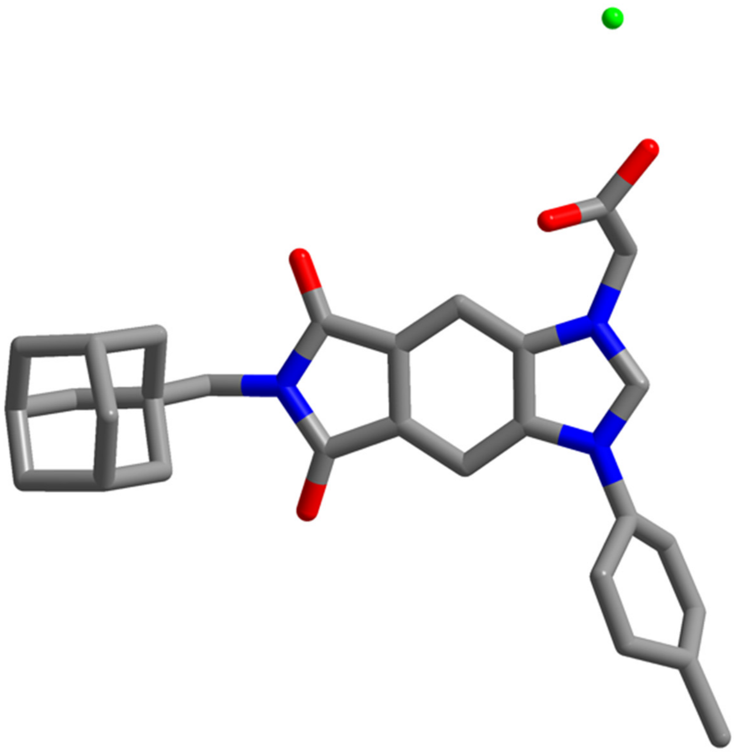

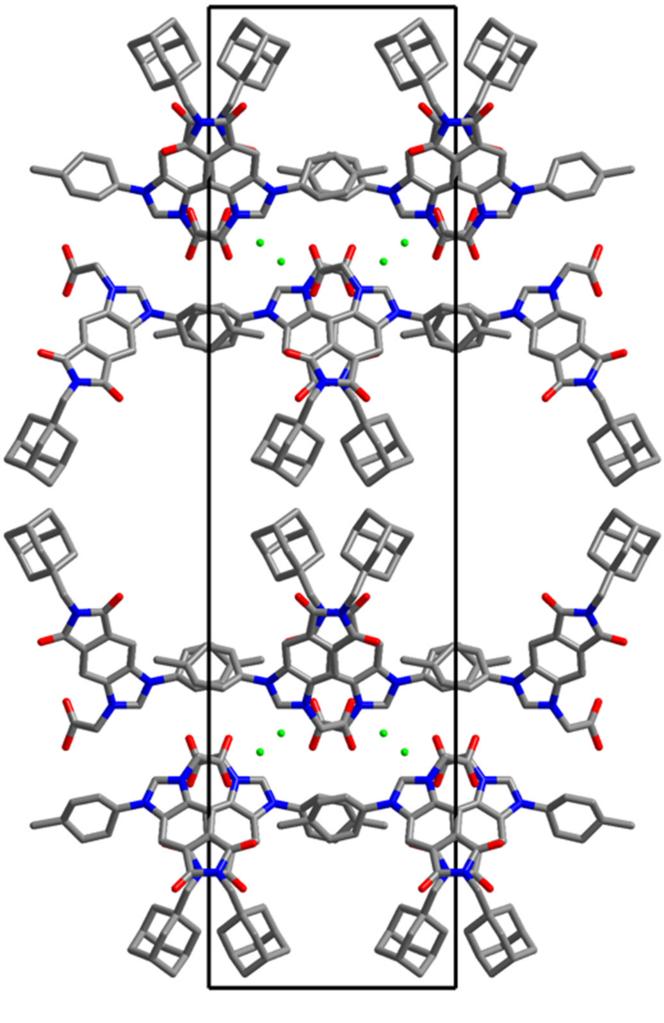

Table S8. Sample and crystal data for D8_2953_HK_BTolBr.

**Identification code**	D8_2953_HK_BTolBr	
**Chemical formula**	$C_{31.50}H_{36}BrN_3O_4$	
**Formula weight**	600.54 g/mol	
**Temperature**	173(2) K	
**Wavelength**	0.71073 Å	
**Crystal size**	0.051 x 0.202 x 0.265 mm	
**Crystal system**	orthorhombic	
**Space group**	I b a 2	
**Unit cell dimensions**	a = 12.5299(12) Å	α = 90°
	b = 49.749(5) Å	β = 90°
	c = 10.0582(9) Å	γ = 90°
**Volume**	6269.8(10) $Å^3$	
**Z**	8	
**Density (calculated)**	1.272 g/cm^3	
**Absorption coefficient**	1.349 mm^{-1}	
**F(000)**	2504	

Table S9. Data collection and structure refinement for D8_2953_HK_BTolBr.

**Theta range for data collection**	2.46 to 25.50°
**Index ranges**	-15<=h<=14, -60<=k<=60, -12<=l<=12
**Reflections collected**	38548
**Independent reflections**	5780 [R(int) = 0.0839]
**Max. and min. transmission**	1.0000 and 0.7241
**Structure solution technique**	direct methods
**Structure solution program**	SHELXT-2014 (Sheldrick 2014)
**Refinement method**	Full-matrix least-squares on F^2
**Refinement program**	SHELXL-2014 (Sheldrick 2014)

**Function minimized**	Σ w(F_o^2 - F_c^2)^2		
**Data / restraints / parameters**	5780 / 85 / 357		
**Goodness-of-fit on F^2**	1.104		
**Δ/σ_{max}**	0.001		
**Final R indices**	4770 data; I>2σ(I)	R1 = 0.0648, wR2 = 0.1614	
	all data	R1 = 0.0790, wR2 = 0.1740	
**Weighting scheme**	w=1/[σ^2(F_o^2)+(0.0760P)^2+30.9620P]<br>where P=(F_o^2+2F_c^2)/3		
**Absolute structure parameter**	0.1(0)		
**Extinction coefficient**	0.0030(5)		
**Largest diff. peak and hole**	0.825 and -0.594 eÅ^{-3}		
**R.M.S. deviation from mean**	0.103 eÅ^{-3}		

Table S10. Atomic coordinates and equivalent isotropic atomic displacement parameters (Å^2) for D8_2953_HK_BTolBr.

U(eq) is defined as one third of the trace of the orthogonalized U_{ij} tensor.

	**x/a**	**y/b**	**z/c**	**U(eq)**
Br1	0.79258(5)	0.75963(2)	0.02132(15)	0.0368(3)
O1	0.3630(8)	0.6013(2)	0.6900(18)	0.126(6)
O2	0.6791(6)	0.64733(17)	0.6389(9)	0.065(2)
O3	0.5771(6)	0.70831(16)	0.2019(8)	0.055(2)
O4	0.5797(5)	0.75415(14)	0.1838(6)	0.0428(15)
N1	0.2400(5)	0.68841(16)	0.3679(8)	0.0391(17)
N2	0.3899(6)	0.71179(15)	0.3555(7)	0.0309(17)
N3	0.5363(8)	0.6179(2)	0.6719(15)	0.086(4)
C1	0.2836(6)	0.71171(17)	0.3284(8)	0.0314(17)
C2	0.3187(6)	0.6732(2)	0.4316(10)	0.042(2)
C3	0.4150(7)	0.68812(17)	0.4190(9)	0.0348(19)
C4	0.5110(6)	0.67932(18)	0.4756(8)	0.036(2)

	x/a	y/b	z/c	U(eq)
C5	0.5044(7)	0.65528(18)	0.5421(15)	0.050(3)
C6	0.4068(8)	0.6409(2)	0.5580(15)	0.073(5)
C7	0.3098(7)	0.6495(2)	0.4978(18)	0.070(5)
C8	0.4264(11)	0.6168(3)	0.6419(19)	0.094(5)
C9	0.5855(8)	0.6407(2)	0.6195(13)	0.057(3)
C10	0.5882(12)	0.5995(4)	0.768(2)	0.108(7)
C11	0.6322(11)	0.5746(3)	0.691(3)	0.123(8)
C12	0.7190(11)	0.5819(3)	0.582(2)	0.107(7)
C13	0.7688(13)	0.5572(3)	0.539(5)	0.169(6)
C14	0.6831(15)	0.5418(4)	0.455(4)	0.173(7)
C15	0.5950(13)	0.5330(3)	0.548(4)	0.167(6)
C16	0.5471(11)	0.5579(3)	0.615(3)	0.154(7)
C17	0.6823(14)	0.5549(4)	0.789(4)	0.168(7)
C18	0.8145(15)	0.5388(4)	0.628(4)	0.174(7)
C19	0.6459(15)	0.5146(4)	0.665(4)	0.179(7)
C20	0.7304(15)	0.5303(4)	0.726(4)	0.172(6)
C21	0.1259(6)	0.6824(2)	0.3579(9)	0.040(2)
C22	0.0914(19)	0.6743(7)	0.253(3)	0.060(5)
C23	0.9783(16)	0.6709(5)	0.238(3)	0.059(5)
C22B	0.0920(16)	0.6575(6)	0.287(3)	0.061(5)
C23B	0.9886(19)	0.6514(5)	0.285(3)	0.063(5)
C24	0.9099(8)	0.6711(3)	0.3375(13)	0.067(4)
C25	0.9476(9)	0.6890(2)	0.4226(18)	0.079(5)
C26	0.0536(9)	0.6944(3)	0.4342(18)	0.087(5)
C27	0.7904(8)	0.6656(5)	0.3281(19)	0.120(8)
C28	0.4556(8)	0.7357(2)	0.3324(10)	0.032(2)
C29	0.5435(8)	0.7304(2)	0.2313(10)	0.036(2)

Table S11. Bond lengths (Å) for D8_2953_HK_BTolBr.

O1-C8	1.208(15)	O2-C9	1.234(12)
O3-C29	1.212(13)	O4-C29	1.355(12)
O4-H4	0.84	N1-C1	1.341(11)
N1-C2	1.397(12)	N1-C21	1.464(11)
N2-C1	1.359(11)	N2-C3	1.376(12)
N2-C28	1.467(12)	N3-C9	1.395(14)
N3-C8	1.411(17)	N3-C10	1.479(18)
C1-H1	0.95	C2-C7	1.361(15)
C2-C3	1.422(12)	C3-C4	1.402(12)
C4-C5	1.373(14)	C4-H4A	0.95
C5-C6	1.426(13)	C5-C9	1.472(15)
C6-C7	1.422(16)	C6-C8	1.489(17)
C7-H7	0.95	C10-C11	1.56(3)
C10-H10A	0.99	C10-H10B	0.99
C11-C17	1.52(3)	C11-C16	1.55(3)
C11-C12	1.59(3)	C12-C13	1.45(3)
C12-H12A	0.99	C12-H12B	0.99
C13-C18	1.40(4)	C13-C14	1.57(4)
C13-H13	1.0	C14-C15	1.51(4)
C14-H14A	0.99	C14-H14B	0.99
C15-C16	1.53(3)	C15-C19	1.62(4)
C15-H15	1.0	C16-H16A	0.99
C16-H16B	0.99	C17-C20	1.50(4)
C17-H17A	0.99	C17-H17B	0.99
C18-C20	1.51(4)	C18-H18A	0.99
C18-H18B	0.99	C19-C20	1.45(3)
C19-H19A	0.99	C19-H19B	0.99
C20-H20	1.0	C21-C22	1.21(3)

C21-C26	1.330(15)	C21-C22B	1.49(3)
C22-C23	1.43(3)	C22-H22	0.95
C23-C24	1.32(3)	C23-H23	0.95
C22B-C23B	1.33(3)	C22B-H22B	0.95
C23B-C24	1.49(3)	C23B-H23B	0.95
C24-C25	1.321(19)	C24-C27	1.525(14)
C25-C26	1.361(16)	C25-H25	0.95
C26-H26	0.95	C27-H27A	0.98
C27-H27B	0.98	C27-H27C	0.98
C28-C29	1.522(12)	C28-H28A	0.99
C28-H28B	0.99		

Table S12. Bond angles (°) for D8_2953_HK_BTolBr.

C29-O4-H4	109.5	C1-N1-C2	108.4(7)
C1-N1-C21	123.7(7)	C2-N1-C21	127.5(7)
C1-N2-C3	108.3(7)	C1-N2-C28	121.4(8)
C3-N2-C28	129.8(8)	C9-N3-C8	112.5(9)
C9-N3-C10	123.7(11)	C8-N3-C10	123.0(11)
N1-C1-N2	110.0(7)	N1-C1-H1	125.0
N2-C1-H1	125.0	C7-C2-N1	129.5(8)
C7-C2-C3	124.4(9)	N1-C2-C3	106.0(8)
N2-C3-C4	130.7(8)	N2-C3-C2	107.0(8)
C4-C3-C2	122.0(8)	C5-C4-C3	114.7(8)
C5-C4-H4A	122.6	C3-C4-H4A	122.6
C4-C5-C6	122.8(9)	C4-C5-C9	130.2(8)
C6-C5-C9	106.7(9)	C7-C6-C5	122.4(10)
C7-C6-C8	128.6(10)	C5-C6-C8	109.0(10)
C2-C7-C6	113.5(8)	C2-C7-H7	123.3
C6-C7-H7	123.3	O1-C8-N3	125.5(13)

O1-C8-C6	129.3(13)	N3-C8-C6	104.6(10)
O2-C9-N3	125.4(10)	O2-C9-C5	127.4(10)
N3-C9-C5	107.2(8)	N3-C10-C11	108.9(17)
N3-C10-H10A	109.9	C11-C10-H10A	109.9
N3-C10-H10B	109.9	C11-C10-H10B	109.9
H10A-C10-H10B	108.3	C17-C11-C16	104.9(14)
C17-C11-C10	110.(2)	C16-C11-C10	115.2(14)
C17-C11-C12	108.1(15)	C16-C11-C12	105.(2)
C10-C11-C12	113.5(12)	C13-C12-C11	107.8(18)
C13-C12-H12A	110.1	C11-C12-H12A	110.1
C13-C12-H12B	110.1	C11-C12-H12B	110.1
H12A-C12-H12B	108.5	C18-C13-C12	123.(4)
C18-C13-C14	107.5(17)	C12-C13-C14	106.4(14)
C18-C13-H13	106.3	C12-C13-H13	106.3
C14-C13-H13	106.3	C15-C14-C13	108.(3)
C15-C14-H14A	110.1	C13-C14-H14A	110.1
C15-C14-H14B	110.1	C13-C14-H14B	110.1
H14A-C14-H14B	108.4	C14-C15-C16	108.9(15)
C14-C15-C19	108.9(16)	C16-C15-C19	107.(3)
C14-C15-H15	110.8	C16-C15-H15	110.8
C19-C15-H15	110.8	C15-C16-C11	112.4(14)
C15-C16-H16A	109.1	C11-C16-H16A	109.1
C15-C16-H16B	109.1	C11-C16-H16B	109.1
H16A-C16-H16B	107.9	C20-C17-C11	115.(3)
C20-C17-H17A	108.6	C11-C17-H17A	108.6
C20-C17-H17B	108.6	C11-C17-H17B	108.6
H17A-C17-H17B	107.5	C13-C18-C20	108.3(19)
C13-C18-H18A	110.0	C20-C18-H18A	110.0
C13-C18-H18B	110.0	C20-C18-H18B	110.0

H18A-C18-H18B	108.4	C20-C19-C15	107.0(17)
C20-C19-H19A	110.3	C15-C19-H19A	110.3
C20-C19-H19B	110.3	C15-C19-H19B	110.3
H19A-C19-H19B	108.6	C19-C20-C17	108.7(17)
C19-C20-C18	113.(3)	C17-C20-C18	109.2(17)
C19-C20-H20	108.8	C17-C20-H20	108.8
C18-C20-H20	108.8	C22-C21-C26	114.1(15)
C22-C21-N1	118.5(14)	C26-C21-N1	122.2(10)
C26-C21-C22B	117.1(11)	N1-C21-C22B	118.8(10)
C21-C22-C23	119.(2)	C21-C22-H22	120.5
C23-C22-H22	120.5	C24-C23-C22	124.(2)
C24-C23-H23	117.9	C22-C23-H23	117.9
C23B-C22B-C21	118.4(19)	C23B-C22B-H22B	120.8
C21-C22B-H22B	120.8	C22B-C23B-C24	119.(2)
C22B-C23B-H23B	120.4	C24-C23B-H23B	120.4
C23-C24-C25	105.4(15)	C25-C24-C23B	116.0(14)
C23-C24-C27	126.1(16)	C25-C24-C27	120.7(13)
C23B-C24-C27	120.7(16)	C24-C25-C26	122.5(12)
C24-C25-H25	118.8	C26-C25-H25	118.7
C21-C26-C25	121.8(13)	C21-C26-H26	119.1
C25-C26-H26	119.1	C24-C27-H27A	109.5
C24-C27-H27B	109.5	H27A-C27-H27B	109.5
C24-C27-H27C	109.5	H27A-C27-H27C	109.5
H27B-C27-H27C	109.5	N2-C28-C29	111.7(9)
N2-C28-H28A	109.3	C29-C28-H28A	109.3
N2-C28-H28B	109.3	C29-C28-H28B	109.3
H28A-C28-H28B	107.9	O3-C29-O4	126.0(10)
O3-C29-C28	125.0(10)	O4-C29-C28	109.0(10)

Table S13. Anisotropic atomic displacement parameters (Å²) for D8_2953_HK_BTolBr.

The anisotropic atomic displacement factor exponent takes the form: $-2\pi^2[h^2 a^{*2} U_{11} + ... + 2 h k a^* b^* U_{12}]$

	U_{11}	U_{22}	U_{33}	U_{23}	U_{13}	U_{12}
Br1	0.0264(4)	0.0577(5)	0.0263(4)	-0.0020(6)	0.0065(5)	-0.0052(3)
O1	0.047(5)	0.116(8)	0.216(16)	0.087(10)	0.003(7)	-0.011(5)
O2	0.041(4)	0.076(5)	0.079(6)	0.013(4)	-0.022(4)	-0.008(4)
O3	0.057(5)	0.055(5)	0.052(5)	-0.016(4)	0.019(4)	-0.010(4)
O4	0.026(3)	0.069(4)	0.034(3)	-0.002(3)	0.004(3)	-0.014(3)
N1	0.020(3)	0.055(4)	0.042(4)	0.007(4)	0.005(3)	0.005(3)
N2	0.030(4)	0.042(4)	0.021(4)	-0.001(3)	0.004(3)	-0.006(3)
N3	0.046(5)	0.076(7)	0.137(11)	0.057(7)	-0.015(6)	-0.008(5)
C1	0.024(4)	0.042(5)	0.029(4)	0.008(3)	0.000(3)	-0.001(3)
C2	0.013(4)	0.060(6)	0.055(6)	0.005(5)	-0.001(4)	-0.005(4)
C3	0.025(4)	0.045(5)	0.035(5)	-0.003(4)	0.009(3)	-0.003(4)
C4	0.019(4)	0.053(5)	0.036(5)	-0.005(3)	0.004(3)	-0.004(3)
C5	0.022(4)	0.048(5)	0.079(9)	0.007(5)	0.005(5)	-0.002(3)
C6	0.037(5)	0.064(7)	0.117(14)	0.034(7)	-0.002(6)	-0.002(5)
C7	0.020(4)	0.065(6)	0.125(15)	0.039(9)	-0.001(6)	-0.012(4)
C8	0.062(8)	0.082(9)	0.137(14)	0.067(10)	-0.006(8)	-0.016(7)
C9	0.035(5)	0.054(6)	0.082(8)	0.020(5)	-0.006(5)	-0.005(4)
C10	0.053(8)	0.112(13)	0.158(18)	0.074(12)	-0.001(10)	0.002(8)
C11	0.042(7)	0.092(11)	0.24(2)	0.073(14)	-0.032(11)	-0.019(7)
C12	0.061(9)	0.078(9)	0.18(2)	0.024(10)	-0.019(10)	-0.007(7)
C13	0.071(7)	0.080(8)	0.355(19)	-0.008(12)	-0.040(12)	0.008(6)
C14	0.077(8)	0.081(8)	0.360(19)	-0.006(11)	-0.043(11)	0.015(7)
C15	0.066(6)	0.074(7)	0.360(18)	0.026(10)	-0.051(10)	-0.003(6)
C16	0.046(7)	0.073(8)	0.34(2)	0.042(11)	-0.049(10)	-0.009(6)
C17	0.071(9)	0.090(9)	0.34(2)	0.095(12)	-0.062(12)	-0.012(8)

	U_{11}	U_{22}	U_{33}	U_{23}	U_{13}	U_{12}
C18	0.069(8)	0.098(9)	0.353(19)	0.020(11)	-0.055(11)	0.009(7)
C19	0.081(8)	0.082(8)	0.374(18)	0.038(11)	-0.058(11)	-0.007(7)
C20	0.070(7)	0.091(8)	0.355(18)	0.058(10)	-0.067(10)	-0.011(7)
C21	0.022(4)	0.064(6)	0.034(5)	0.011(4)	-0.005(4)	0.003(4)
C22	0.021(7)	0.076(10)	0.084(12)	-0.010(10)	0.015(8)	-0.009(9)
C23	0.023(8)	0.075(10)	0.081(12)	-0.008(9)	0.015(7)	-0.014(8)
C22B	0.029(7)	0.070(10)	0.083(11)	-0.020(9)	0.016(7)	-0.017(8)
C23B	0.035(7)	0.071(9)	0.083(11)	-0.017(9)	0.010(7)	-0.018(8)
C24	0.016(5)	0.123(11)	0.062(8)	-0.003(8)	0.009(5)	0.005(6)
C25	0.041(6)	0.051(6)	0.145(14)	-0.027(8)	0.032(7)	0.001(5)
C26	0.032(6)	0.087(9)	0.141(14)	-0.046(9)	0.022(7)	0.000(6)
C27	0.016(5)	0.24(2)	0.105(13)	-0.026(14)	-0.001(6)	-0.023(8)
C28	0.024(4)	0.051(6)	0.020(4)	0.003(4)	-0.006(4)	-0.006(4)
C29	0.028(5)	0.049(5)	0.029(5)	-0.003(5)	-0.006(4)	-0.012(4)

Table S14. Hydrogen atomic coordinates and isotropic atomic displacement parameters (Å^2) for D8_2953_HK_BTolBr.

	x/a	y/b	z/c	U(eq)
H4	0.6284	0.7513	0.1280	0.064
H1	0.2457	0.7260	0.2875	0.038
H4A	0.5757	0.6892	0.4684	0.043
H7	0.2452	0.6396	0.5034	0.084
H10A	0.5359	0.5936	0.8356	0.129
H10B	0.6473	0.6089	0.8136	0.129
H12A	0.6848	0.5911	0.5061	0.128
H12B	0.7731	0.5942	0.6207	0.128
H13	0.8266	0.5625	0.4759	0.202
H14A	0.6540	0.5537	0.3853	0.207
H14B	0.7159	0.5259	0.4121	0.207
H15	0.5389	0.5228	0.4983	0.2
H16A	0.4909	0.5521	0.6783	0.185
H16B	0.5132	0.5693	0.5468	0.185
H17A	0.7387	0.5645	0.8391	0.201
H17B	0.6271	0.5492	0.8530	0.201
H18A	0.8755	0.5471	0.6746	0.208
H18B	0.8410	0.5229	0.5782	0.208
H19A	0.5907	0.5101	0.7322	0.215
H19B	0.6749	0.4977	0.6283	0.215
H20	0.7649	0.5193	0.7973	0.207
H22	0.1384	0.6704	0.1812	0.072
H23	-0.0490	0.6684	0.1506	0.071
H22B	0.1429	0.6463	0.2445	0.073
H23B	-0.0348	0.6347	0.2496	0.076

	x/a	y/b	z/c	U(eq)
H25	-0.1013	0.6984	0.4778	0.095
H26	0.0766	0.7071	0.4986	0.104
H27A	-0.2391	0.6634	0.4177	0.18
H27B	-0.2216	0.6492	0.2768	0.18
H27C	-0.2448	0.6808	0.2838	0.18
H28A	0.4881	0.7415	0.4174	0.038
H28B	0.4097	0.7505	0.2999	0.038
